# Production of Stevia‐Based Persimmon Fruit Leather by Microwave Oven and Its Optimization With Response Surface Methodolog

**DOI:** 10.1002/fsn3.70036

**Published:** 2025-02-14

**Authors:** Muhammad Hamza Alam, Muhammad Haseeb Ahmad, Muhammad Imran, Misbah Ur Rehman, Muhammad Imran Khan, Muhammad Kamran Khan, Waseem Khalid, Sulaiman Ali Alharbi, Hossam M. Aljawdah, Felix Kwashie Madilo

**Affiliations:** ^1^ Department of Food Science, Faculty of Life Sciences Government College University Faisalabad Pakistan; ^2^ Department of Mathematics and Statistics, Faculty of Sciences University of Agriculture Faisalabad Pakistan; ^3^ Department of Organic Chemistry, Faculty of Chemical Sciences and Technologies University of Castilla La Mancha Ciudad Real Spain; ^4^ University Institute of Food Science and Technology The University of Lahore Lahore Pakistan; ^5^ Department of Botany and Microbiology, College of Science King Saud University Riyadh Saudi Arabia; ^6^ Department of Zoology, College of Science King Saud University Riyadh Saudi Arabia; ^7^ Department of Food Science and Technology Ho Technical University Ho Ghana

**Keywords:** microwave drying, moisture content, persimmon fruit leather, sensory analysis, stevia, texture

## Abstract

The consumer preference for low‐calorie and nutritious foods has urged the attention of researchers and food designers to develop food products with alternative organic ingredients. For the first time, persimmon is successfully developed into fruit leather (pestil) using a non‐caloric stevia sweetener. The study started with the production of persimmon ( *Diospyros kaki* ) leather, initially evaluating the compatibility of non‐caloric stevia (*S*_T_) sweetener with hydrocolloids (corn starch, pectin, and guar gum) by the hot air oven method, in which guar gum exhibited superior flexibility. After setting the leather recipe, the trials were performed in a microwave oven by employing a Box–Behnken experimental design to optimize the drying process. Independent variables including microwave power (MP), microwave time (MT), and leather thickness (LT) have shown their optimum values at 210 W, 30 min, and 3.5 mm, respectively, based on physicochemical analyses focusing on moisture content, texture, color, total phenolic content (TPC), and antioxidant capacity (AC). MP had the most substantial impact on the variables, followed by MT, while LT showed the least influence. Optimized development of microwave products emphasized better physicochemical attributes, highlighting the energy‐efficient nature of the resulting product in comparison to hot air‐dried product. Sensory evaluation favored the optimized microwave‐dried product over the hot air oven leather products. Therefore, utilizing novel processing technologies like microwave drying is recommended for producing functional (*S*_T_) based persimmon leather to uphold superior product quality.

## Graphical abstract

Response surface methodology provided the optimum values of microwave power (210 W), microwave time (30 min), and leather thickness (3.5 mm) for microwave drying of low‐calorie nutritive persimmon fruit leather. Optimized development of microwave products emphasized better physicochemical attributes, highlighting the energy‐efficient nature of the resulting product in comparison to hot air‐dried products. Preference mapping via sensory evaluation favored the optimized microwave‐dried product over the hot air oven leather product.

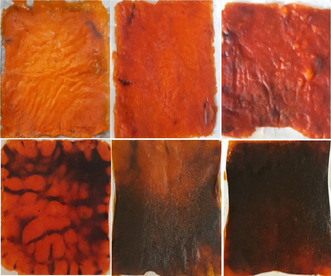

**Abbreviations**
(η_conv_): energy efficiency for convective dryer or hot air drier (%)
(η_mwd_): energy efficiency of microwave drying system (%)
∆𝑇: temperature difference (K)
*A*
: drying tray (𝑚^2^)
a*: redness
AC: antioxidant capacity
Adeq_pre_
: adequacy of precision
ANOVA: analysis of variance
b*: yellowness
BBD: Box‐Behnken design
CH: cohesiveness
CN: chewiness
*C*_𝑎_
: specific heat capacity of inlet air (kJ kg^−1^ K^−1^)
DPPH: 2,2‐diphenyl‐1‐Picrylhydrazyl
*E*_T_
: total energy consumption (kWh)
*E*_TH_
: thermal energy (J)
GAE: gallic acid equivalent
GU: gumminess
HA: hardness
L*: lightness
LT: leather thickness
MP: microwave power
MT: microwave time
*m*_w_
: mass of water evaporated from the sample (kg)
*P*
: microwave input power (kW)
*P*_out_
: total electric power output of the dryer (kWh)
*P*_T_
: total electric power (kWh)
*R* ^2^
: coefficient of determination
RSM: response surface methodology
SD: standard deviation
SG: springiness
SGs: steviol glycosides
*S*_T_
: stevia
*t*
: dying time (h)
*T*_amb_
: ambient air temperature (°C)
*T*_i_
: temperature of the drying medium at the inlet of the dryer (°C)
*T*_o_
: temperature of the drying medium at the outlet of the dryer (°C)
TPC: total phenolic content
*λ*_w_
: latent heat of vaporization of water (kJ kg^−1^)
𝑣: drying air speed (ms^−1^)
𝜌𝑎: air density (kgm^−3^)

## 1 Introduction

Persimmon ( *Diospyros kaki* L.) is a climacteric fruit with unique sensory qualities and a nutritional profile that belongs to the *Ebanaceae* family. Regarding macronutrients, persimmon has a low protein and fat content and around 16% carbohydrates, mainly sugars. These sugars are mostly fructose, glucose, and sucrose. They are rich in micronutrients, especially provitamin A (beta‐carotene) and vitamin C, and provide significant potassium. However, they contain lower levels of magnesium and phosphorus. Their functional properties are linked to bioactive compounds and antioxidant capacity, including vitamin C, phenolic acids, flavonoids, tannins, phloridzin, and carotenoids. Persimmon is a perishable fruit with a limited shelf life (Nasr et al. 2022; Pérez‐Burillo et al. 2018) and its season is rather brief, lasting no longer than 2–3 months. Fruit post‐storage degeneration and extreme softening are major issues in the fruit industry. These problems lead to significant postharvest losses, ranging from 40% to 50% in developing countries to 10%–12% in developed countries (Khan et al. 2023). In this situation, industries need novel alternatives like fruit leather (pestil). This helps to avoid losses connected with persimmon fruit handling and season. Fruit leathers are a unique method of fruit preservation that has emerged as a viable way to capitalize on fruit oversupply by adding value and broadening fruit‐eating replacements. Initially, these fruit snacks were homemade solutions for preserving fruit. Recently, they have evolved into commercially manufactured and nutritionally valuable products in the food industry (Concha‐Meyer et al. 2016). Fruit leather is one example of a product that can be developed as a result of dehydration (Das et al. 2019). The drying of fruit leather is usually done in the sun or in the shade, while other drying methods include oven drying, microwave drying, and infrared drying (Tontul and Topuz 2017). A successful drying technology is judged by how well it reduces moisture content to a specific level. It should also minimize quality deterioration during the drying process (Pu and Sun 2017). Recent research efforts in the literature are primarily directed toward advancing the development of cost‐effective, dependable, efficient, and environmentally friendly microwave components (Acar et al. 2022). Microwaves are part of the electromagnetic spectrum, traveling at the speed of light. They have wavelengths from 1 m to 1 mm, corresponding to a frequency range of 300 MHz–300 GHz. Microwave heating offers several advantages, including selective material heating, rapid heating, and non‐contact application. It allows for quick start‐up and shutdown, along with in situ waste treatment. The equipment used is portable, versatile, and suitable for various applications (Sun et al. 2016). Very few studies have been conducted to manufacture the fruit leathers using microwave as drying technology, for instance, mango ( *Mangifera indica* ) leather (Pushpa et al. 2006), apricot ( *Prunus armeniaca* L.) leather (Suna et al. 2014), and medlar ( *Mespilus germanica* ) leather (Suna 2019). Only two studies have focused on producing persimmon fruit leather. One of these studies used tray drying (Dursun and Dalgıç 2018). In another study, a mixture of mango and persimmon was developed into leather using microwave heating (Mohamed et al. 2018). While no study has yet been reported on the manufacturing of persimmon leather with non‐caloric sweeteners like *S*_T_.

*Stevia rebaudiana* Bertoni, which is a shrub from the Asteraceae family native to northeast Paraguay. It produces non‐caloric sweetening compounds called steviol glycosides (SGs). There are over 30 different known SGs. The most common ones are stevioside and rebaudioside‐A. These two compounds account for about 90% of the sweet glycosides found in stevia leaves (Goksel et al. 2013). *S*_T_ is a natural sweetener with a low caloric content and a sweetness level 200–300 times greater than sucrose. It is a promising substitute for sucrose and has several beneficial effects on specific physiological systems. These include cardiovascular and renal benefits. They also help reduce blood sugar, radionuclide, and cholesterol levels. Additionally, these benefits enhance cell regeneration and improve blood coagulation. They inhibit neoplastic growth and strengthen blood vessels. Notably, they have significant antioxidant properties (Rodriguez Furlán et al. 2016). There are different ways of incorporating SGs into foods, e.g., as a highly purified rebaudioside in powder or as a mixture (Karp et al. 2016). SGs have typical amphiphilic structures. They consist of a hydrophobic diterpenoid or steviol backbone. This backbone is attached to various hydrophilic sugar residues at the C_13_ and C_19_ positions. Because of this amphiphilicity, SGs can self‐assemble into micelles when in aqueous solutions. This property makes them a promising delivery system for hydrophobic active substances (Yang et al. 2022). The acceleration of micelle formation under microwave irradiation might be due to reaction selectivity. Additionally, the trans‐glycosylation phenomenon plays a role. This process causes the generation of reducing sugar intermediates. These intermediates are then followed by the disproportionation of steviol from SGs (Ko et al. 2021).

In this study, persimmon fruit leather was produced using *S*_T_ under an experimental design in order to optimize the microwave drying process parameters by analyzing moisture content, color, texture profile, TPC, and AC. In addition to these, the sensory characteristics were also analyzed between the optimized microwave drying method and hot air oven drying method to evaluate the acceptability of the product.

## 2 Materials and Methods

### 2.1 Raw Materials

Persimmon ( *Diospyros kaki* ) fruits were purchased from a local retail outlet in Faisalabad, Pakistan (31.4187° N, 73.0791° E) during the last week of October 2023. Various additives like citric acid, color (orange red), maltodextrin, pectin, corn starch, and guar gum were obtained from a local supplier (Hafeez Rungwala, Montgomery Bazar, Faisalabad, Pakistan). Additionally, stevia powder was procured online from Micro Ingredients (California, USA). For chemical analysis, sodium carbonate (Na_2_CO_3_), Folin–Ciocalteu reagent, 2,2‐diphenyl‐1‐picrylhydrazyl (DPPH), gallic acid, formic acid, and methanol were purchased from Merck (Beijing, China). All chemicals and reagents used were of analytical grade.

### 2.2 Preparation and Storage of Persimmon Fruit Pulp

The fruits were sorted and washed with chlorinated water to remove any contaminants. They were then peeled using a stainless‐steel knife. After peeling, the fruits were pulped using a blender (Kenwood—HB682, United Kingdom). The total soluble solids of the pulp were measured in Brix degrees using a refractometer (Fisherbrand Handheld Analog Brix/Sucrose Refractometer, Fisher Scientific, USA). Soluble solid content (°Brix) of the pulp was 18°B. The pulp was put into an airtight plastic container and stored at −18°C in a refrigerator (Dawlance, Pakistan) for further processing.

### 2.3 Preliminary Treatment Plan

Three treatments with different thickening agents were performed to get the best formulation by hot air oven drying method (Table 1). Preliminary tests for the formulation of edible persimmon leathers and their sensory acceptance evaluations on the characteristics of flexibility, color, and taste were carried out by a panel of semi‐trained professionals.

**TABLE 1:** Preliminary treatment plan for persimmon leather containing *S*_T_ by hot air oven method.

Treatments	Pulp (g)	Water (mL)	Color (g)	Maltodextrin (g)	Citric acid (g)	Stevia (g)	Thickeners (g)		
							Corn Starch	Pectin	Guar gum
T_1_	80	20	0.1	0.1	0.5	0.1	2.5	—	—
T_2_	80	20	0.1	0.1	0.5	0.1	—	1	—
T_3_	80	20	0.1	0.1	0.5	0.1	—	—	0.8

### 2.4 Preparation of Persimmon Leather

The pulp in the plastic box container was thawed for 1 h at room temperature. The pulp was combined with distilled water, color, maltodextrin, citric acid, *S*_T_, and thickener. This mixture was then cooked in an open kettle for 5–8 min. Stirring was kept constant during cooking. The mixture was heated until the Brix level reached 30°B–35°B. The total soluble solids of the pulp were determined in Brix degrees using a refractometer (Fisherbrand Handheld Analog Brix/Sucrose Refractometer, Fisher Scientific, USA). Afterwards, the mixture (approximately 40–50 g) was evenly spread to a depth of 5–8 mm. For hot air drying, the mixture was spread on a stainless‐steel tray. For microwave drying (ANEX, AG‐9039, Pakistan), it was spread on a microwavable plate. The samples were then subjected to their respective drying methods until the fruit leather was formed.

#### 2.4.1 Conventional Hot Air Oven Drying

The hot air drying was conducted using an oven (Memmert, UN110, Schwabach, Germany) at a constant 15% relative humidity (RH) and 60°C. Every 40 min, the sample was weighed (WT2002A, Changzhou, China) and returned to the dryer. The hot air drying process took 5.5 h. to complete, resulting in leather with 14%–15% moisture content. The leather was then placed in a zip‐lock bag to prevent moisture exchange. It was stored at room temperature until analysis.

### 2.5 Experimental Design and Statistical Analysis for Microwave Drying

A Response Surface Methodology (RSM) Box–Behnken design (BBD) was used to optimize microwave drying for persimmon leather. STATGRAPHICS software (Version 5.1, 2000, Statistical Graphics Corporation, USA) was employed to develop mathematical models and analyze the influence of independent variables on dependent ones. The following polynomial model was used in this study given in Equation (1):

(1) $$Y=\beta_{0}+\sum_{i=1}^{\infty }\beta_{i}X_{i}+\sum_{i=1}^{\infty }\beta_{\mathrm{ii}}X_{i}^{2}\;+\sum_{i<j}^{\infty }\beta_{\mathrm{ij}}X_{i}X_{j}$$

Where *Y* represents the predicted response, *X*_*i*_ and *X*_*j*_ represent the independent variables and *b*_o_, *b*_*i*_, *b*_*ii*_ and *b*_*ij*_ represent the intercept, linear, quadratic and interaction regression coefficients of the model respectively. In this study, the key factors were microwave power (MP), leather thickness (LT), and microwave time (MT). The three independent factors were investigated at three levels: 0 was the midpoint to determine the experimental error, while +1 and −1 were used for high and low levels. The variables coded as −1, 0, and +1, which correspond to specific values for each factor. For MP, the decoded values are 90, 180, and 270 W. For LT, the values are 1, 3, and 5 mm. Finally, for MT, the decoded times are 20, 40, and 60 min (Table 2).

**TABLE 2:** The experimental range and levels of independent variables for the Box–Behnken design (BBD) applied to persimmon leather containing *S*_T_.

Parameters	Real values of the coded level		
	Low (−1)	Center (0)	High (1)
MP	90	180	270
MT	20	40	60
LT	1	3	5

Abbreviations: LT, leather thickness; MP, microwave power; MT, microwave time.

The BBD was further processed to check the significance of the studied parameters by applying the analysis of variance (ANOVA). The standard error of the design was calculated on the basis of the central points replicated thrice in the experimentation. Moreover, *R* ^2^ (the coefficient of determination) was calculated to see how well the data fit the regression model. Furthermore, the adequacy of the fitted polynomial model was also expressed by the adjusted *R* ^2^, adequacy precision (Adeq_pre_).

### 2.6 Moisture Content

Halogen moisture analyzer (Model A100MA, ACE, Ravenna, USA), which has readability of 0.001 g had been used to determine the moisture content of persimmon fruit leathers. A 1 g sample was placed in the sample pan. The temperature was then rapidly increased and held at 120°C using a halogen lamp. The measurements were completed in a time period ranging from 3 to 12 min in this test condition. The halogen lamp of the moisture analyzer heated the sample in the chamber until it reached a constant mass. The moisture contents of all samples were measured in triplicate.

### 2.7 Color

Color analyses of persimmon fruit leathers were carried out by a high quality colorimeter (CR400, Konica Minolta, Chiyoda TKY, Japan) using CIE Color lab space equipped with illuminant D65. Color was determined in terms of L* (lightness), a* [chromaticity (redness and greenness)], and b* [chromaticity (yellowness and blueness)]. Hue angle and chroma were calculated from these values. Chroma changes from 0 (dull) to 60 (vivid) and was calculated from a* and b* values by using the following Equation (2):

(2) $$\text{Chroma}={\left(a^{*2}+b^{*2}\right)}^{1/2}$$

Hue angle, h*, is the color value and is defined as starting at +a* axis. It is expressed in degrees: 0° (red), 90° (yellow), 180° (green) and 270° (blue). h* was also calculated from a* and b* values by using the following Equation (3):

(3) $$\mathrm{Hue}\;\text{angle}=\arctan\;\left(b^{*}/a^{*}\right)$$

Calibration of the instrument was done using black and white tiles. The light source used was D65 (noon daylight). The color parameters of all samples were measured in triplicate.

### 2.8 Texture Profile Analysis

The texture profile analysis (TPA) was performed using a texture analyzer (TA‐XT plus, Stable Micro Systems, Surrey, United Kingdom). The texture analyzer was attached to the computer software “Exponent Connect” (Texture Technologies, Hamilton, MA, USA). Fruit leather samples measured 10.16 × 10.16 cm in area. The samples had thicknesses of 1, 3, and 5 mm. They were placed on a flat surface, and the upper compression platen was lowered onto them. The TPA test consisted of two cycles of compression. In each cycle, the persimmon fruit leather sample was penetrated by a cylinder probe. The test was done at room temperature. Hardness (HA), springiness (SG), cohesiveness (CH), gumminess (GM), and chewiness (CN) attributes were measured using a probe compressed twice, and a force–time graph was generated. The probe used was a cylindrical needle P/2. The duration between the first and second compressions was 5 s. HA, CH, SG, GM, and CN were calculated using Equations (4), (5), (6), (7) and (8) (Wu et al. 2014):

(4) HA=Height of the 1st peak

(5) CH=Second positive area/First positive area

(6) SG=Time of 2nd bite/Time of 1st bite

(7) GM=HA×CH

(8) CN=GU×SG

The texture parameters of all samples were measured in triplicate. The TPA settings were as given in Table 3.

**TABLE 3:** TA‐XT plus instrument settings for persimmon leather containing *S*_T_.

Parameters	Values
Software	Exponent Connect
Mode	Compression
Pre‐test speed	1 mm s^−1^
Test speed	8 mm s^−1^
Post‐test speed	8 mm s^−1^
Target mode	Strain
Strain	10%
Trigger Type	Force
Trigger force	20 g

### 2.9 Total Phenolic Content

The phenolic content was measured using the Folin–Ciocalteu assay by UV‐vis mass spectrophotometer as reported by (Singleton and Rossi 1965) with some modifications. The extraction method involved dissolving a 2 g sample in a 20 mL solution. This solution was composed of 75% methanol and 0.1% formic acid. The mixture was then sonicated by a sonication machine (VCX750, Newtown, USA) for 5 min at 50% amplitude and filtered using Whatman paper No. 1. The reaction mixture consisted of sample extract or standard (100 μL), Folin reagent (125 μL, 0.2 N), methanol (1525 μL), and sodium carbonate solution (1250 μL, 7.5% w/v) were added making up 3 mL volume into test tube. It is then vortexed (XH‐D, Wincom, Changsha, China) for 10 s and incubated for 30 min at room temperature in a dark cabin. Absorbance of samples and blank was measured at 765 nm using a UV–Visible spectrophotometer (SPECORD 200 Plus, Jena, Germany). The TPC results are stated in mg gallic acid equivalent per gram dry matter of the sample (mg GAE g^−1^ DM). A standard curve was prepared in duplicate by making a standard solution of 10 mg mL^−1^ gallic acid diluted to final concentrations of 0, 200, 400, 600, 800, and 1000 μg gallic acid mL^−1^. The standard gallic acid calibration curve was developed by plotting the linear regression curve of absorbance versus gallic acid concentration (mg mL^−1^). TPC was expressed as mg of GAE/g of extract and was calculated using the following Equation (9):

(9) TPC=C×V/W

where C = gallic acid equivalent concentration obtained from the calibration curve (mg mL^−1^), *V* = volume of the stock solution of extract (mL), *W* = dry weight of the extract found in the stock solution (g), and TPC = total phenolic content expressed as mg of GAE g^−1^ dry extract. The TPC of all samples was measured in triplicate.

### 2.10 Antioxidant Capacity by DPPH Analysis

The DPPH (2,2‐diphenyl‐1‐picrylhydrazyl radical (DPPH•, DPPH‐R)) was discovered by Goldsmith and Renn in 1922. It is known for its stability and is widely used in antioxidant assays. DPPH has a high redox potential, which allows it to oxidize most common natural antioxidants. Because of these properties, it is commonly used to assay the antioxidant capacity of biological materials (Flieger and Flieger 2020). Its activity of scavenging free radicals of the extracts was assessed by following the modified procedure of (Mimica‐Dukic et al. 2004). Briefly, a small amount (100 μL) of the sample extract was mixed in 2 mL of 0.1 mM DPPH solution in methanol and kept in the dark at ambient temperature for 1 h. The absorbance of the sample and control was determined at 517 nm using a UV–Visible spectrophotometer (SPECORD 200 Plus, Germany). Results were expressed as % DPPH calculated according to following Equation (10):

(10) $$\text{DPPH}\;\left(\%\right)=\left(A_{c}–A_{s}/A_{c}\right)\times 100$$

where *A*_s_ and *A*_c_ are absorbance of sample and control, respectively. The antioxidant capacity of all samples was measured in triplicate.

### 2.11 Energy Consumption of Microwave and Conventional Methods

Energy consumption by the microwave oven is given in Equation (11) (Kassem et al. 2011; Motevali, Minaei, and Khoshtagaza 2011).

(11) $$E_{T}=P\times t$$

where *E*_T_ represents total energy consumption (kW h), *P* is the microwave input power (kW) and *t* is the drying time (h).

In a hot air dryer, *E*_T_ was estimated from the sum of total electric power (*P*_T_) and thermal energy (*E*_TH_). *P*_T_ can be obtained as the product of the total electric power output of the dryer (*P*_out_) and the total drying time (dt). *E*_T_, *P*_T_, and *E*_TH_ required to execute drying are obtained using Equation (12), (13), and (14) (Chibuike et al. 2021).

(12) $$E_{T}=P_{T}+E_{\mathrm{TH}}$$

(13) $$P_{T}=\mathrm{dt}\times P_{\text{out}}$$

(14) $$E_{\mathrm{TH}}={\text{(34𝑣·34𝑣𝐴·34𝑣𝐴𝜌}}_{\text{34𝑣𝐴𝜌𝑎}}\cdot C_{\text{34𝑎}}\text{·∆34𝑎𝑇)·}t$$

where 𝑣, *A*, *C*_𝑎_, and 𝜌_𝑎_ are the drying air speed (ms^−1^), area of the drying tray (𝑚^2^), specific heat capacity of inlet air (kJ kg^−1^ K^−1^) and air density (kgm^−3^). ∆𝑇 is the difference in temperature (K) between the ambient air and the hot drying air and t is the drying time. *C*_𝑎_ and 𝜌_𝑎_ can be calculated using Equation (15) and (16) (Beigi 2016).

(15) $${\text{34𝑎𝑇𝜌}}_{\text{34𝑎𝑇𝜌𝑎}}=\frac{101.325}{\;0.287\text{Tabs}}$$

(16) $$C_{a}=1.04841-\frac{3.8371\text{Tabs}}{10^{4}}+\frac{9.43578T^{2}\mathrm{abs}\;}{10^{7}}-\frac{5.4903T^{3}\mathrm{abs}}{10^{10}}+\frac{7.9209T^{4}\mathrm{abs}}{10^{14}}$$

### 2.12 Energy Efficiency of Microwave and Conventional Methods

Energy efficiency of microwave drying system (η_mwd_) was calculated as the ratio of heat energy utilized for evaporating water from the sample to the heat supplied by the dryer given in Equation (17) (Soysal et al. 2006; Surendhar et al. 2019; Yilmaz et al. 2021).

(17) $$\eta_{\mathrm{mwd}}=\frac{m_{W}\times \lambda_{w}}{P\times t}\times 100$$

where *m*_w_ is the mass of water (kg) evaporated from the sample, *λ*_w_ is the latent heat of vaporization of water (2257 kJ kg^−1^), *P* is the MP level (W) and t is the drying time (s). The energy efficiency for convective dryers(η_conv_) or hot air dryer is calculated using the temperatures of the drying medium and ambient air in Equation (18) (Menon et al. 2020; Vieira et al. 2007).

(18) $$\eta_{\text{conv}}=\frac{T_{i}-T_{o}}{T_{i}-T_{\mathrm{amb}}}$$

where *T*_*i*_ is the temperature of the drying medium at the inlet of the dryer, *T*_o_ is the temperature of the drying medium at the outlet of the dryer, and *T*_amb_ is the ambient air temperature.

### 2.13 Sensory Evaluation

The sensory analysis of two persimmon fruit leathers was conducted using a 9‐point hedonic scale. This scale ranged from 1, indicating “disliked extremely,” to 9, indicating “liked extremely.” The comparison was made between hot air oven and microwave drying methods (Gámbaro and McSweeney 2020). One product was from the hot air oven drying method, and the other one was from the microwave drying having stevia as the main sweetener. A panel of food professionals bearing an age between 20 and 30 years and having food technology background was selected for the descriptive sensory evaluation. The samples presented in a completely randomized order, and water was provided between samples during analysis. Panelists were asked to record their ratings for sensory quality attributes sweetness, flavor, color, appearance, chewiness, hardness, stickiness, and overall acceptability of the experimented samples, and the highest score of each treatment was selected. Sensory data analysis was performed using Minitab Statistical (v.17) to test the hypothesis that the means of both treatments are the same at 5% level of significance. It was also assumed that the variances of both treatments for each attribute (or parameter) are equal. The *t*‐test for equality of two independent sample means was applied.

## 3 Results

### 3.1 Moisture Content

**FIGURE 1:**
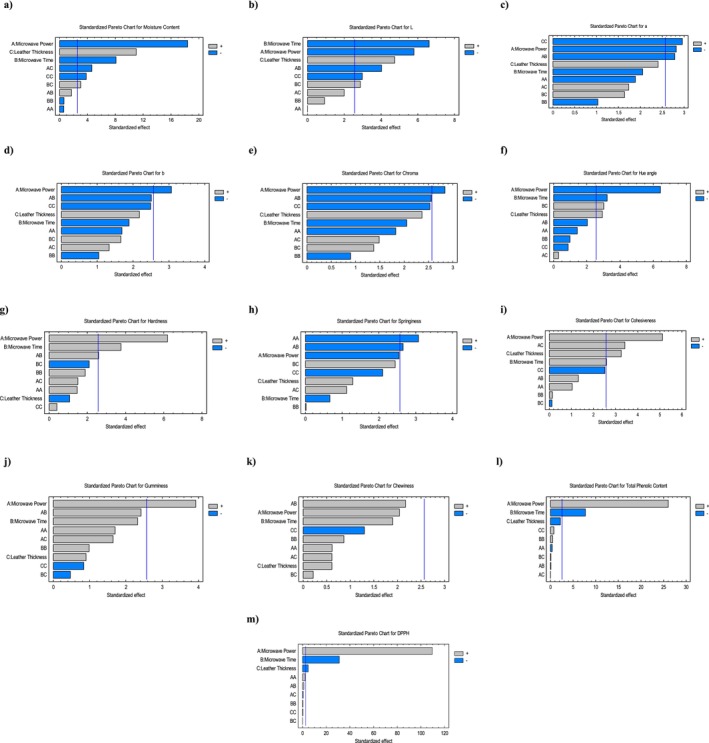
Persimmon fruit leather (*S*_T_) pareto charts. (a) moisture content, (b) L* value, (c) a* value, (d) b* value, (e) chroma, (f) hue angle, (g) hardness, (h) springiness, (i) cohesiveness, (j) gumminess, (k) chewiness, (l) TPC, and (m) DPPH.

**FIGURE 2:**
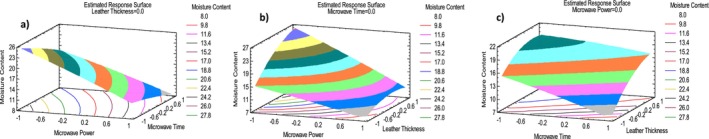
3D Response surface plots of persimmon leather containing *S*_T_ representing the effect of mutual interactions of studied parameters on the moisture content. Interaction between microwave power (MP) and microwave time (MT) (a), microwave power (MP) and leather thickness (LT) (b), and microwave time (MT) and leather thickness (LT) (c), while keeping third parameter at central value.

Table 4 summarizes the final values of moisture content. The highest moisture (26.76%) was in run 12 (90 W, 5 mm thickness, 40 min), while the lowest (7.01%) was in run 7 (270 W, 1 mm, 40 min). Tables 5 and 6 demonstrate ANOVA analysis and the adequacy of model for moisture content. The moisture content model was highly significant (*p* ≤ 0.001). The *p*‐values of the A, B, and C of the linear term and AC of interaction term were lower than 0.001 making it highly significant while *C* ^2^ of quadratic term was only significant. In this study, *R* ^2^ was found to be 99.13% and adjusted *R* ^2^ was 97.57%. This means that the model explains 99.13% of the variation in the experimental data. Adeq_pre_ determines the signal‐to‐noise ratio. Adeq_pre_ ratio for moisture content was > 4, signifying an adequate signal, model efficacy and can be used to navigate the design space. Table 7 shows regression coefficients for moisture content. The negative coefficient values (−5.84, −2.57) for moisture content indicate that when MP and MT increased, moisture content values would decrease. The quadratic regression equation describing the effect of the process variables on the moisture content is reported in Table 8. Lastly, optimal parameters (194.53 W, 41.87 min, 2.62 mm) achieved the desirable moisture content of 15% illustrated in Table 9, respectively. Figure 1a presents the standardized Pareto chart, illustrating the linear, quadratic, and interaction effects of each factor studied on the moisture content of persimmon leather. All bars outside the vertical line are statistically significant at the chosen significance level. According to this figure, MP is the most significant factor followed by the LT and MT. The regression coefficient is used to generate a response surface plot of the regression model. Figure 2 illustrates the 3D response surface of moisture content versus MP, MT, and LT. According to Figure 2, MP had a greater effect on moisture content than LT and MT. Furthermore, moisture content showed indirect relation with MP and MT, and a direct relation with LT.

**TABLE 4:** Box–Behnken design matrix and analysis results for moisture content, color, texture profile attributes, TPC, and DPPH activity of persimmon leather containing *S*_T_.

Runs	Independent variables			Response variables												
	MP (W)	MT (min)	LT (mm)	Moisture content (%)	Color parameters					Texture parameters					TPC	DPPH
					L*	a*	b*	Chroma	Hue angle	HA (g)	SG (%)	CH (%)	GU (g)	CN (g)	mg GAE g^−1^	%
1	90	40	1	15.55	37.19	23.37	22.92	32.73	44.44	110.88	290.65	18.3	20.29	58.98	0.042	48.05
2	270	40	5	9.88	29.18	12.48	10.01	16.00	38.73	288.86	103.34	73.9	213.47	220.6	0.122	71.24
3	180	20	5	18.98	39.79	21.01	19.32	31.37	42.82	120.99	310.09	30.6	37.02	114.81	0.092	62.11
4 cp	180	40	3	16.89	34.87	25.13	23.34	34.30	42.89	111.71	351.66	37.9	42.34	148.89	0.080	59.44
5	180	20	1	14.76	37.11	16.17	15.19	22.19	43.21	105.33	320.92	18.6	19.59	62.87	0.097	63.24
6 cp	180	40	3	16.88	34.85	25.15	23.37	34.33	42.90	111.85	351.34	38.1	42.61	149.7	0.081	59.45
7	270	40	1	7.01	14.38	2.01	1.49	2.50	36.55	198.67	55.67	16.5	32.78	18.25	0.133	72.47
8 cp	180	40	3	16.86	34.82	25.23	23.45	34.44	42.91	111.99	351.09	38.2	42.78	150.2	0.082	59.47
9	270	20	3	13.26	45.68	26.63	24.13	35.94	42.18	199.65	270.34	42.2	84.25	80.33	0.146	75.12
10	270	60	3	8.65	19.19	6.73	4.99	8.38	36.56	483.55	95.34	78.7	380.56	1029.74	0.111	68.29
11	90	20	3	25.49	41.31	18.12	17.21	24.99	43.53	49.59	185.14	19.1	9.47	17.54	0.051	51.83
12	90	40	5	26.76	40.01	18.65	19.22	26.78	45.86	47.89	175.44	17.4	8.33	14.62	0.031	47.21
13	90	60	3	17.79	38.97	22.63	21.11	30.95	43.01	69.97	394.39	33.2	23.23	91.62	0.017	44.28
14	180	60	1	7.88	14.87	3.99	2.98	4.98	36.75	330.87	59.23	25.5	84.37	49.97	0.076	56.81
15	180	60	5	17.54	34.76	23.15	22.31	32.15	43.94	132.77	401.43	35.4	47	188.68	0.072	55.73

Abbreviations: a*, redness; b*, yellowness; CH, cohesiveness; CN, chewiness; cp, central point; GU, gumminess; HA, hardness; L*, lightness; LT, leather thickness; MP, microwave power; MT, microwave time; SG, Springiness; TPC, total phenolic content.

**TABLE 5:** ANOVA of response variables of persimmon leather containing *S*_T_.

Result source	*p*												
	Moisture content	Color parameters					Texture parameters					TPC	DPPH
	(%)	L*	a*	b*	Chroma	Hue angle	HA (g)	SG (%)	CH (%)	GU (g)	CN (g)	mg GAE g^−1^	%
Model	0.0001**	0.0039*	0.0491*	0.0652	0.0640	0.0146*	0.0167*	0.0708	0.0213*	0.0741	0.2648	< 0.0001**	< 0.0001**
Linear	0.0000**	0.0010**	0.0416*	0.0426*	0.0419*	0.0032*	0.0042*	0.1439	0.0067*	0.0290*	0.1541	0.0000**	0.0000**
A: MP	0.0000**	0.0022*	0.0372*	0.0279*	0.0363*	0.0014*	0.0016*	0.0517	0.0037*	0.0111*	0.0962	0.0000**	0.0000**
B: MT	0.0005**	0.0012*	0.0953	0.1184	0.0956	0.0231*	0.0131*	0.5357	0.0491*	0.0676	0.1156	0.0006**	0.0000**
C: LT	0.0001**	0.0051*	0.0615	0.0814	0.0642	0.0326*	0.3301	0.2540	0.0226*	0.4099	0.5677	0.0819	0.0045*
Interactions	0.0117*	0.0166*	0.0700	0.1008	0.1037	0.0709	0.0706	0.0632	0.0699	0.1394	0.2795	0.9992	0.6484
AB	0.1465	0.0100*	0.0386*	0.0539	0.0509	0.0979	0.0489*	0.0452*	0.2458	0.0605	0.0821	0.9249	0.2961
AC	0.0057**	0.1021	0.1434	0.2408	0.1982	0.7743	0.1927	0.3117	0.0188*	0.1607	0.5674	1.0000	0.5553
BC	0.0293*	0.0349*	0.1630	0.1588	0.2285	0.0294*	0.0897	0.0588	0.9067	0.6593	0.8381	0.9249	0.9386
Quadratic	0.0587	0.1093	0.0850	0.1339	0.1252	0.4282	0.2670	0.0730	0.1610	0.3074	0.4602	0.8073	0.2802
A^2^	0.5698	0.9996	0.1181	0.1523	0.1275	0.2127	0.2012	0.0276*	0.3455	0.1499	0.5649	0.7189	0.0823
B^2^	0.5568	0.3977	0.3508	0.3446	0.4109	0.3670	0.1184	0.9841	0.8948	0.3692	0.4274	0.6541	0.6464
C^2^	0.0123*	0.0306*	0.0316*	0.0554	0.0527	0.4209	0.7013	0.0894	0.0534	0.4385	0.2512	0.4806	0.7277

Abbreviations: a*, redness; b*, yellowness; CH, cohesiveness; CN, chewiness; GU, gumminess; HA, hardness; L*, lightness; LT, leather thickness; MP, microwave power; MT, microwave time; NS, non‐significant (> 0.05); SG, springiness; TPC, total phenolic content.

* Significant (≤ 0.05).

** Highly significant (≤ 0.001).

**TABLE 6:** Adequacy of model for persimmon leather containing *S*_T_.

Fitting statistics	Response variables												
	Moisture content	L*	a*	b*	Chroma	Hue angle	HA	SG	CH	GU	CN	TPC	DPPH
*R* ^2^ (%)	99.1352	96.5158	89.6615	88.2356	88.34	93.9041	93.5464	87.7855	92.8383	87.5273	76.5756	99.3299	99.9616
Adjusted *R* ^2^ (%)	97.5785	90.2442	71.0522	67.0597	67.352	82.9315	81.93	65.7995	79.9472	65.0763	34.4116	98.1238	99.8926
Adeq_pre_	25.6956	11.4833	6.5908	6.0552	6.1067	9.3679	10.9135	6.1406	9.0108	7.6687	5.4110	29.1935	121.9564
SD	0.8995	2.9970	4.3799	4.59188	6.55750	1.2551	50.8924	72.4023	8.5205	58.517	201.609	0.0050	0.3086
Mean	15.61	33.13	18.03	16.74	24.80	41.75	164.97	247.74	34.91	72.54	159.79	0.0822	59.65

Abbreviations: a*, redness; Adeq_pre_, adequacy of precision; b*, yellowness; CH, cohesiveness; CN, chewiness; GU, gumminess; HA, hardness; L*, lightness; *R* ^2^, coefficient of determination; SD, standard deviation; SG, springiness; TPC, total phenolic content.

**TABLE 7:** Regression coefficients for response variables of persimmon leather containing *S*_T_.

Results source	Regression coefficients												
	Moisture content	L*	a*	b*	Chroma	Hue angle	HA	SG	CH	GU	CN	TPC	DPPH
Intercept	16.8767	34.8467	25.17	23.3867	34.3567	42.9	111.85	351.363	38.0667	42.5767	149.597	0.081	59.4533
MP: A	−5.8488	−6.1313	−4.365	−4.98	−6.5788	−2.8525	111.55	−65.1162	15.4125	81.2175	145.77	0.0464	11.9687
MT: B	−2.5788	−7.0125	−3.1788	−3.0575	−4.7538	−1.435	67.7	−17.0125	7.7875	48.1038	135.557	−0.0138	−3.3988
LT: C	3.495	5.0238	3.7188	3.535	5.4875	1.3	−19.405	32.9787	9.8	18.5988	43.58	−0.0039	−0.535
AB	0.7725	−6.0375	−6.1025	−5.76	−8.38	−1.275	65.88	−96.0625	5.6	70.6375	218.832	−0.0003	0.18
AC	−2.085	2.995	3.7975	3.055	4.8625	0.19	38.295	40.72	14.575	48.1625	61.6775	0.0	−0.0975
BC	1.36	4.3025	3.58	3.8	4.4975	1.895	−53.44	88.2575	−0.525	−13.7	21.6925	0.0003	0.0125
*A* ^2^	−0.2846	−0.0008	−4.2975	−4.0333	−6.2308	−0.9325	38.9625	−115.852	4.6167	51.7617	64.6204	−0.001	0.3483
*B* ^2^	−0.2946	1.4417	−2.345	−2.4933	−3.0608	−0.6475	49.8775	0.7908	0.6167	30.0392	90.5904	0.0013	0.0783
*C* ^2^	−1.7921	−4.6558	−6.745	−5.9433	−8.6233	−0.5725	10.7625	−79.2367	−11.1583	−25.6208	−136.105	0.002	−0.0592

Abbreviations: a*, redness; b*, yellowness; CH, cohesiveness; CN, chewiness; GU, gumminess; HA, hardness; L*, lightness; LT, leather thickness; MP, microwave power; MT, microwave time; SG, springiness; TPC, total phenolic content.

**TABLE 8:** Regression equations for dependent and independent variables in persimmon leather containing *S*_T_.

Variable	Regression equations
Moisture content	16.8767 − 5.84875*MP − 2.57875*MT + 3.495*LT − 0.284583*MP^2^ + 0.7725*MP*MT − 2.085*MP*LT − 0.294583*MT^2^ + 1.36*MT*LT − 1.79208*LT^2^
L* value	34.8467 − 6.13125*MP − 7.0125*MT + 5.02375*LT − 0.000833333*MP^2^ − 6.0375*MP*MT + 2.995*MP*LT + 1.44167*MT^2^ + 4.3025*MT*LT − 4.65583*LT^2^
a* value	25.17 − 4.365*MP − 3.17875*MT + 3.71875*LT − 4.2975*MP^2^ – 6.1025*MP*MT + 3.7975*MP*LT‐ 2.345*MT^2^ + 3.58*MT*LT − 6.745*LT^2^
b* value	23.3867 − 4.98*MP − 3.0575*MT + 3.535*LT − 4.03333*MP^2^ − 5.76*MP*MT + 3.055*MP*LT − 2.49333*MT^2^ + 3.8*MT*LT − 5.94333*LT^2^
Chroma value	34.3567 − 6.57875*MP − 4.75375*MT + 5.4875*LT − 6.23083*MP^2^ – 8.38*MP*MT + 4.8625*MP*LT − 3.06083*MT^2^ + 4.4975*MT*LT‐ 8.62333*LT^2^
Hue angle	42.9 − 2.8525*MP − 1.435*MT + 1.3*LT − 0.9325*MP^2^ − 1.275*MP*MT + 0.19*MP*LT − 0.6475*MT^2^ + 1.895*MT*LT − 0.5725*LT^2^
HA	111.85 + 111.55*MP + 67.7*MT − 19.405*LT + 38.9625*MP^2^ + 65.88*MP*MT + 38.295*MP*LT + 49.8775*MT^2^ − 53.44*MT*LT + 10.7625*LT^2^
SG	351.363 − 65.1162*MP − 17.0125*MT + 32.9787*LT − 115.852*MP^2^ − 96.0625*MP*MT + 40.72*MP*LT + 0.790833*MT^2^ + 88.2575*MT*LT − 79.2367*LT^2^
CH	38.0667 + 15.4125*MP + 7.7875*MT + 9.8*LT + 4.61667*MP^2^ + 5.6*MP*MT + 14.575*MP*LT+ 0.616667*MT^2^ − 0.525*MT*LT‐ 11.1583*LT^2^
GU	42.5767 + 81.2175*MP + 48.1038*MT + 18.5988*LT + 51.7617*MP^2^ + 70.6375*MP*MT+ 48.1625*MP*LT + 30.0392*MT^2^ − 13.7*MT*LT‐ 25.6208*LT^2^
CN	149.597 + 145.77*MP + 135.557*MT + 43.58*LT + 64.6204*MP^2^ + 218.832*MP*MT + 61.6775*MP*LT + 90.5904*MT^2^ + 21.6925*MT*LT − 136.105*LT^2^
TPC	0.081 + 0.046375*MP − 0.01375*MT − 0.003875*LT − 0.001*MP^2^ − 0.00025*MP*MT + 0.0*MP*LT + 0.00125*MT^2^ + 0.00025*MT*LT + 0.002*LT^2^
DPPH	59.4533 + 11.9687*MP − 3.39875*MT − 0.535*LT + 0.348333*MP^2^ + 0.18*MP*MT − 0.0975*MP*LT + 0.0783333*MT^2^ + 0.0125*MT*LT − 0.0591667*LT^2^

Abbreviations: a*, redness; b*, yellowness; CH, cohesiveness; CN, chewiness; GU, gumminess; HA, hardness; L*, lightness; LT, leather thickness; MP, microwave power; MT, microwave time; SG, springiness; TPC, total phenolic content.

**TABLE 9:** Optimized response of persimmon leather containing *S*_T_.

Responses	Desirability	Values	Conditions		
			MP	MT	LT
Moisture content	Maintaining at 15%	—	194.53	41.87	2.92
L*	Maximum	43.94	270	20.01	3.79
a*	Maximum	26.42	128.92	43.75	3.33
b*	Maximum	25.42	90	55.14	3.56
Chroma	Maximum	36.38	153.49	39.04	3.34
Hue angle	Maximum	46.44	90	59.84	5
HA	Maintaining at 69 g	—	148.86	39.33	3.53
SG	Maintaining at 394%	—	109.62	56.62	3.50
CH	Maintaining at 33%	—	141.48	41.53	3.26
GU	Maintaining at 23 g	—	154.92	39.04	3.25
CN	Maintaining at 91 g	—	137.17	38.52	3.35
TPC (mg GAE g^−1^)	Maximum	0.148	269.76	20	1
DPPH (%)	Maximum	75.61	269.70	20	1.01

Abbreviations: a*, redness; b*, yellowness; CH, cohesiveness; CN, chewiness; GU, gumminess; HA, hardness; L*, lightness; LT, leather thickness; MP, microwave power; MT, microwave time; SG, springiness; TPC, total phenolic content.

### 3.2 Color

#### 3.2.1 L* Value

**FIGURE 3:**
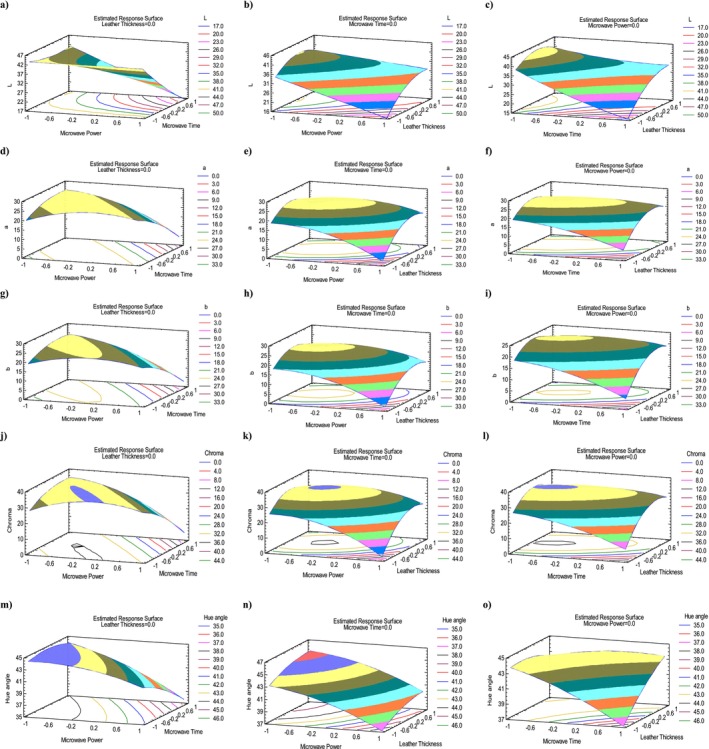
3D Response surface plots of persimmon leather containing *S*_T_ representing the effect of mutual interactions of studied parameters on the color parameters. L* (a–c); a* (d–f); b* (g–i); chroma (j–l); and hue angle (m–o). Interaction between microwave power (MP) and microwave time (MT) (a, d, g, j, m); microwave power (MP) and leather thickness (LT) (b, e, h, k, n); and microwave time (MT) and leather thickness (LT) (c, f, i, l, o), while keeping third parameter at central value.

Table 4 shows the final values of the L* value of persimmon fruit leather. Highest L* (45.68) was in run 9 (270 W, 3 mm thickness, 20 min), while the lowest (14.38) was in run 7 (270 W, 1 mm, 40 min). Tables 5 and 6 demonstrate ANOVA analysis and the adequacy of model for lightness (L*). The L* model was significant (*p* ≤ 0.05). The *p*‐values of the A, B, and C of the linear term and BC, AC of interaction term and *C* ^2^ of quadratic term were statistically significant. In this study, *R* ^2^ was found to be 96.51% and adjusted *R* ^2^ was 90.24%. This means that the model explains 96.51% of the variation in the experimental data. Adeq_pre_ ratio for L* was > 4, signifying an adequate signal, model efficacy and can be used to navigate the design space. Table 7 shows regression coefficients for L*. The negative coefficient values (−7.01, −6.13) for L* indicates that when MP and MT increased, L* values would decrease. The quadratic regression equation describing the effect of the process variables on the L* is reported in Table 8. Lastly, optimal parameters (270 W, 20.01 min, 3.79 mm) achieved the maximum L* value of 43.94 according to desirability illustrated in Table 9, respectively. Figure 1b presents the standardized Pareto chart, illustrating the linear, quadratic, and interaction effects of each factor studied on the L* of persimmon leather. According to this figure, MT is the most significant factor, followed by the MP and LT. Figure 3a–c illustrates the 3D response surface of L* versus MP, MT, and LT. According to Figure 3a–c, MT had a greater effect on L* than MP and LT. Furthermore, L* showed an indirect relation with MP and MT and a direct relation with LT, just like moisture content.

#### 3.2.2 a* Value

Table 4 shows the final values of a* value of persimmon fruit leather. Highest redness (26.63) was in run 9 (270 W, 3 mm thickness, 20 min), while lowest (2.01) was in run 7 (270 W, 1 mm, 40 min). Tables 5 and 6 demonstrates ANOVA analysis and adequacy of model for redness (a*). The a* model was significant (*p* ≤ 0.05). The *p*‐values of the A, B, C of linear term and BC, AC of interaction term and *C* ^2^ of quadratic term were statistically significant. In this study, *R* ^2^ was found to be 89.66% and adjusted *R* ^2^ was 71.05%. This means that the model explains 89.66% of the variation in the experimental data. Adeq_pre_ ratio for a* was > 4, signifying an adequate signal, model efficacy and can be used to navigate the design space. Table 7 shows regression coefficients for a*. The negative coefficient values (−4.36, −3.17) for a* indicates that when MP and MT increased, a* values would decrease. The quadratic regression equation describing the effect of the process variables on the a* is reported in Table 8. Lastly, optimal parameters (128.92 W, 43.75 min, 3.33 mm) achieved the maximum a* value of 26.42 according to desirability illustrated in Table 9, respectively. Figure 1c presents the standardized Pareto chart, illustrating the linear, quadratic, and interaction effects of each factor studied on the a* of persimmon leather. According to this figure, C^2^ is the most significant factor followed by the MP and AB. Figure 3d–f illustrates the 3D response surface of a* versus MP, MT and LT. According to Figure 3d–f, MP had a greater effect on a* than MT and LT. Furthermore, a* showed indirect relation with MP and MT and direct relation with LT just like L* value.

#### 3.2.3 b* Value

Table 4 presents the final values of b* of persimmon fruit leather. The highest yellowness (24.13) was in run 9 (270 W, 3 mm thickness, 20 min), while the lowest (1.49) was in run 7 (270 W, 1 mm, 40 min). Tables 5 and 6 demonstrate ANOVA analysis and adequacy of model for yellowness (b*). The b* model was non‐significant (*p* > 0.05). The *p*‐value of the A of linear term was only statistically significant. In this study, *R* ^2^ was found to be 88.23%, and adjusted *R* ^2^ was 67.05%. This means that the model explains 88.23% of the variation in the experimental data. Adeq_pre_ ratio for b* was > 4, signifying an adequate signal, model efficacy and can be used to navigate the design space. Table 7 shows regression coefficients for b* value. The negative coefficient values (−4.98, −3.05) for b* indicates that when MP and MT increased, b* values would decrease. The quadratic regression equation describing the effect of the process variables on the b* is reported in Table 8. Lastly, optimal parameter conditions (90 W, 55.14 min, 3.56 mm) achieved the maximum b* value of 25.42 according to desirability illustrated in Table 9, respectively. Figure 1d presents the standardized Pareto chart, illustrating the linear, quadratic, and interaction effects of each factor studied on the b* of persimmon leather. According to this figure, only MP crossed the vertical statistically significant line and showed MP is the most significant factor, followed by the AB and C^2^. Figure 3g–i illustrates the 3D response surface of b* versus MP, MT and LT. According to Figure 3g–i, MP had a greater effect on b* than MT and LT. Furthermore, b* showed an indirect relation with MP and MT and a direct relation with LT, just like a* value.

#### 3.2.4 Chroma Value

Table 4 presents the final values of Chroma of persimmon fruit leather. Highest chroma (35.94) was in run 9 (270 W, 3 mm thickness, 20 min), while lowest (2.5) was in run 7 (270 W, 1 mm, 40 min).

Tables 5 and 6 demonstrate ANOVA analysis and adequacy of the model for chroma. The chroma model was non‐significant (*p* > 0.05). The *p*‐value of the A of the linear term was only statistically significant in the model. In this study, *R* ^2^ was found to be 88.34% and adjusted *R* ^2^ was 67.35%. This means that the model explains 88.34% of the variation in the experimental data. Adeq_pre_ ratio for chroma was > 4, signifying an adequate signal, model efficacy, and can be used to navigate the design space. Table 7 shows regression coefficients for chroma. The negative coefficient values (−6.57, −4.75) for chroma indicates that when MP and MT decrease, chroma values would increase. The quadratic regression equation describing the effect of the process variables on the chroma is reported in Table 8. Lastly, optimal parameters conditions (153.49 W, 35.04 min, 3.34 mm) achieved the maximum chroma value of 36.48 according to desirability illustrated in Table 9, respectively. Figure 1e presents the standardized Pareto chart, illustrating the linear, quadratic, and interaction effects of each factor studied on the chroma of persimmon leather. According to this figure, only MP crossed the vertical statistically significant line and showed MP is the most significant factor, followed by the AB and C^2^. Figure 3j–l illustrates the 3D response surface of chroma versus MP, MT, and LT. According to Figure 3j–l, MP had a greater effect on chroma than MT and LT. Furthermore, chroma showed indirect relation with MP and MT and a direct relation with LT, just like other color parameters.

#### 3.2.5 Hue Angle

Table 4 presents the final values of hue angles of persimmon fruit leather. The highest hue angle (45.84) was in run 12 (90 W, 5 mm thickness, 40 min), while lowest (36.55) was in run 7 (270 W, 1 mm, 40 min). Tables 5 and 6 demonstrate ANOVA analysis and adequacy of model for hue angle. The model for hue angle was significant (*p* ≤ 0.05). The *p*‐values of the A, B, and C of linear term and BC of interaction term were statistically significant. In this study, *R* ^2^ was found to be 93.90%, and adjusted *R* ^2^ was 82.93%. This means that the model explains 93.90% of the variation in the experimental data. Adeq_pre_ ratio for hue angle was > 4, signifying an adequate signal, model efficacy and can be used to navigate the design space. Table 7 shows regression coefficients for hue angle. The negative coefficient values (−2.85, −1.43) for hue angle indicates that when MP and MT increased, hue angle values would decrease. The quadratic regression equation describing the effect of the process variables on the hue angle is reported in Table 8. Lastly, optimal parameters (90 W, 59.84 min, 5 mm) achieved the maximum hue angle value of 46.44 according to desirability illustrated in Table 9, respectively. Figure 1f presents the standardized Pareto chart, illustrating the linear, quadratic, and interaction effects of each factor studied on the hue angle of persimmon leather. According to this figure, MP is the most significant factor, followed by the MT and BC. Figure 3m–o illustrates the 3D response surface of hue angle versus MP, MT, and LT. According to Figure 3m–o, MP had a greater effect on hue angle than MT and LT. Furthermore, hue angle showed an indirect relation with MP and MT and a direct relation with LT, the same as other color parameters.

### 3.3 Texture Profile

#### 3.3.1 Hardness

**FIGURE 4:**
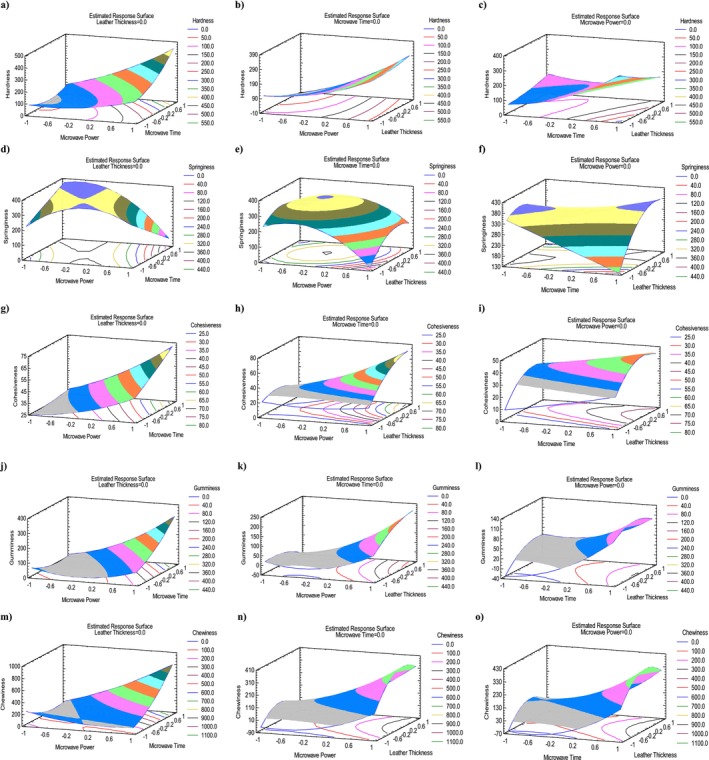
3D response surface plots of persimmon leather containing *S*_T_ representing the effect of mutual interactions of studied parameters on the color parameters. Hardness (a–c); springiness (d–f); cohesiveness (g–i); gumminess (j–l); and chewiness (m–o). Interaction between microwave power (MP) and microwave time (MT) (a, d, g, j, m), microwave power (MP) and leather thickness (LT) (b, e, h, k, n) and microwave time (MT) and leather thickness (LT) (c, f, i, l, o), while keeping third parameter at central value.

Table 4 presents the final values of hardness of persimmon fruit leather. Maximum HA (483.55 g) was in run 12 (270 W, 3 mm thickness, 60 min), while minimum (47.89 g) was in run 12 (90 W, 5 mm, 40 min). Tables 5 and 6 demonstrate ANOVA analysis and adequacy of model for HA. The model for HA was significant (*p* ≤ 0.05). The *p*‐values of the A, B of linear term and AB of interaction term were statistically significant. In this study, *R* ^2^ was found to be 93.54% and adjusted *R* ^2^ was 81.93%. This means that the model explains 93.54% of the variation in the experimental data. Adeq_pre_ ratio for HA was > 4, signifying an adequate signal, model efficacy and can be used to navigate the design space. Table 7 shows regression coefficients for HA. The positive coefficient values (111.55, 67.7) for HA indicates that when MP and MT increased, HA values would increase. The quadratic regression equation describing the effect of the process variables on the HA is reported in Table 8. Lastly, optimal parameters (148.86 W, 39.33 min, 3.53 mm) achieved the desirable HA value of 69 g illustrated in Table 9, respectively. Figure 1g presents the standardized Pareto chart, illustrating the linear, quadratic, and interaction effects of each factor studied on the HA of persimmon leather. According to this figure, MP is the most significant factor, followed by the MT and AB. Figure 4a–c illustrates the 3D response surface of HA versus MP, MT, and LT. According to Figure 4a–c, MP had a greater effect on HA than MT and LT. Furthermore, HA showed a direct relation with MP and MT and an indirect relation with LT.

#### 3.3.2 Springiness

Table 4 presents the final values of SG of persimmon fruit leather. Maximum SG (401.43%) was in run 15 (180 W, 5 mm thickness, 60 min), while minimum (55.67%) was in run 7 (270 W, 1 mm, 40 min). Tables 5 and 6 demonstrate ANOVA analysis and the adequacy of the model for SG. The SG model was non‐significant (*p* > 0.05). The *p*‐value of the AB of interaction term was only statistically significant in the model. In this study, *R* ^2^ was found to be 87.78% and adjusted *R* ^2^ was 65.79%. This means that the model explains 87.78% of the variation in the experimental data. Adeq_pre_ ratio for SG was > 4, signifying an adequate signal, model efficacy and can be used to navigate the design space. Table 7 shows regression coefficients for SG. The negative coefficient values (−66.11, −17.01) for SG indicates that when MP and MT decrease, SG values would increase. The quadratic regression equation describing the effect of the process variables on the SG is reported in Table 8. Lastly, optimal parameters conditions (109.62 W, 56.62 min, 3.5 mm) achieved the desirable SG of 394% illustrated in Table 9, respectively. Figure 1h presents the standardized Pareto chart, illustrating the linear, quadratic, and interaction effects of each factor studied on the SG of persimmon leather. According to this figure, only *A* ^2^ and AB crossed the vertical statistically significant line and showed *A* ^2^ is the most significant factor, followed by the AB and MP. Figure 4d–f illustrates the 3D response surface of SG versus MP, MT, and LT. According to Figure 4d–f, MP had a greater effect on SG than MT and LT. Furthermore, SG showed an indirect relation with MP and MT and a direct relation with LT.

#### 3.3.3 Cohesiveness

Table 4 presents the final values of CH of persimmon fruit leather. Maximum CH (78.7%) was in run 10 (270 W, 3 mm thickness, 60 min), while minimum (17.4%) was in run 12 (90 W, 5 mm, 40 min). Tables 5 and 6 demonstrate ANOVA analysis and the adequacy of the model for CH. The model for CH was significant (*p* ≤ 0.05). The *p*‐values of the A, B, and C of linear term and AC of interaction term were statistically significant. In this study, *R* ^2^ was found to be 92.83%, and adjusted *R* ^2^ was 79.94%. This means that the model explains 92.83% of the variation in the experimental data. Adeq_pre_ ratio for CH was > 4, signifying an adequate signal, model efficacy, and can be used to navigate the design space. Table 7 shows regression coefficients for CH. The positive coefficient values (15.41, 7.78) for CH indicates that when MP and MT increased, CH values would increase. The quadratic regression equation describing the effect of the process variables on the CH is reported in Table 8. Lastly, optimal parameters (141.48 W, 41.53 min, 3.26 mm) achieved the desirable CH value of 33% illustrated in Table 9, respectively. Figure 1i presents the standardized Pareto chart, illustrating the linear, quadratic, and interaction effects of each factor studied on the CH of persimmon leather. According to this figure, MP is the most significant factor, followed by the AC and LT. Figure 4g–i illustrates the 3D response surface of CH versus MP, MT, and LT. According to Figure 4g–i, MP had a greater effect on CH than MT and LT. Furthermore, CH showed a direct relation with MP, MT, and LT.

#### 3.3.4 Gumminess

Table 4 exhibits the final values of GU of persimmon fruit leather. Maximum GU (380.56 g) was in run 10 (270 W, 3 mm thickness, 60 min), while minimum (8.33 g) was in run 12 (90 W, 5 mm, 40 min). Tables 5 and 6 demonstrate ANOVA analysis and the adequacy of the model for GU. The GU model was non‐significant (*p* > 0.05). The *p*‐value of the A of linear term was only statistically significant in the model. In this study, *R* ^2^ was found to be 87.52%, and adjusted *R* ^2^ was 65.07%. This means that the model explains 87.52% of the variation in the experimental data. Adeq_pre_ ratio for GU was > 4, signifying an adequate signal, model efficacy and can be used to navigate the design space. Table 7 shows regression coefficients for GU. The positive coefficient values (81.21, 48.10) for GU indicates that when MP and MT increase, GU values would increase as well. The quadratic regression equation describing the effect of the process variables on the GU is reported in Table 8. Lastly, optimal parameters conditions (154.92 W, 39.04 min, 3.25 mm) achieved the desirable GU of 23 g illustrated in Table 9, respectively. Figure 1j presents the standardized Pareto chart, illustrating the linear, quadratic, and interaction effects of each factor studied on the GU of persimmon leather. According to this figure, only MP crossed the vertical statistically significant line and showed MP is the most significant factor followed by the AB and B. Figure 4j–l illustrates the 3D response surface of GU versus MP, MT, and LT. According to Figure 4j–l, MP had a greater effect on GU than MT and LT. Furthermore, GU showed a direct relation with MP, MT, and LT.

#### 3.3.5 Chewiness

Table 4 presents the final values of CN of persimmon fruit leather. Maximum CN (1029.74 g) was in run 10 (270 W, 3 mm thickness, 60 min), while minimum (55.67 g) was in run 12 (90 W, 5 mm, 40 min). Tables 5 and 6 demonstrate ANOVA analysis and the adequacy of the model for CN. The CN model was non‐significant (*p* > 0.05). The *p*‐values of linear, interaction and quadratic terms were non‐significant in the model as well. In this study, *R* ^2^ was found to be 76.57% and adjusted *R* ^2^ was 34.41%. This means that the model explains 76.57% of the variation in the experimental data. Whereas Adeq_pre_ ratio for CN was > 4, signifying an adequate signal, model efficacy, and can be used to navigate the design space. Table 7 shows regression coefficients for CN. The positive coefficient values (145.77, 135.55) for CN indicates that when MP and MT decrease, CN values would decrease. The quadratic regression equation describing the effect of the process variables on the CN is reported in Table 8. Lastly, optimal parameter conditions (137.17 W, 33.52 min, 3.35 mm) achieved the desirable CN of 91 g illustrated in Table 9, respectively. Figure 1k presents the standardized Pareto chart, illustrating the linear, quadratic, and interaction effects of each factor studied on the CN of persimmon leather. According to this figure, no variable crossed the vertical statistically significant line but the impact of AB was highest, followed by the MP and MT. Figure 4m–o illustrates the 3D response surface of CN versus MP, MT, and LT. According to Figure 4m–o, MP had a greater effect on CN than MT and LT. Furthermore, CN showed a direct relation with MP, MT, and LT.

### 3.4 Total Phenolic Content

**FIGURE 5:**
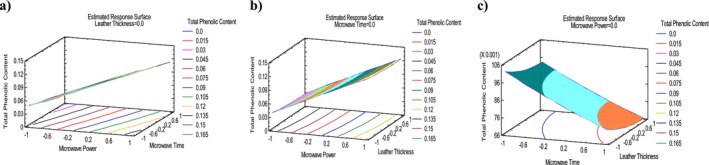
3D Response surface plots of persimmon leather containing *S*_T_ representing the effect of mutual interactions of studied parameters on the TPC. Interaction between microwave power (MP) and microwave time (MT) (a), microwave power (MP) and leather thickness (LT) (b), and microwave time (MT) and leather thickness (LT) (c), while keeping third parameter at central value.

Table 4 shows the final values of TPC. The highest TPC value, 0.146 mg GAE g^−1^, was found in run 9 with 270 W, 3 mm thickness, and 20 min. The lowest TPC value, 0.017 mg GAE g^−1^, occurred in run 13 with 90 W, 3 mm thickness, and 60 min. Tables 5 and 6 demonstrate ANOVA analysis and adequacy of model for TPC. The TPC model was highly significant (*p* ≤ 0.001). The *p*‐values of only A and B of linear term were lower than 0.001 making it highly significant. In this study, *R* ^2^ was found to be 99.32% and adjusted *R* ^2^ was 98.12%. This means that the model explains 99.32% of the variation in the experimental data. Adeq_pre_ ratio for TPC was > 4, signifying an adequate signal, model efficacy, and can be used to navigate the design space. Table 7 shows regression coefficients for TPC. The positive and negative coefficient values (0.0464, −0.0138) for TPC indicates that when MP and MT increased, TPC values would increase for MP and decrease for MT. The quadratic regression equation describing the effect of the process variables on the TPC is reported in Table 8. Lastly, optimal parameters (269.76 W, 20 min, 1 mm) achieved the maximum TPC value of 0.148 mg GAE g^−1^ according to desirability illustrated in Table 9, respectively. Figure 1l presents the standardized Pareto chart, illustrating the linear, quadratic, and interaction effects of each factor studied on the TPC of persimmon leather. According to this figure, only MP and MT crossed the vertical statistically significant line and showed MP is the most significant factor followed by the MT and LT. The regression coefficient is used to generate a response surface plot of the regression model. Figure 5 illustrates the 3D response surface of moisture content versus MP, MT, and LT. According to Figure 5, MP had a greater effect on TPC than LT and MT. Furthermore, TPC showed an indirect relation with MT and LT and a direct relation with MP.

### 3.5 2,2‐Diphenyl‐1‐Picrylhydrazyl (DPPH)

**FIGURE 6:**
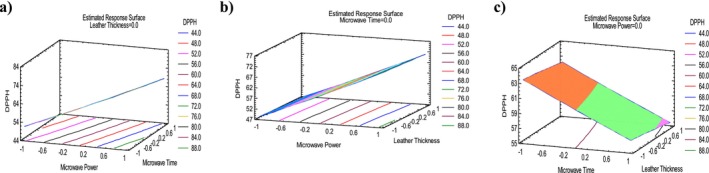
3D Response surface plots of persimmon leather containing *S*_T_ representing the effect of mutual interactions of studied parameters on the DPPH. Interaction between microwave power (MP) and microwave time (MT) (a), microwave power (MP) and leather thickness (LT) (b), and microwave time (MT) and leather thickness (LT) (c), while keeping third parameter at central value.

Table 4 presents the final values of DPPH. The highest DPPH value, 75.12%, was observed in run 9 with 270 W, 3 mm thickness, and 20 min. The lowest value, 44.28%, was recorded in run 13 with 90 W, 3 mm thickness, and 60 min. Tables 5 and 6 demonstrate ANOVA analysis and adequacy of model for DPPH. The DPPH model was highly significant (*p* ≤ 0.001). The *p*‐values of the A, B, and C of linear term were lower than 0.001, making it highly significant. In this study, *R* ^2^ was found to be 99.96% and adjusted *R* ^2^ was 99.89%. This means that the model explains 99.96% of the variation in the experimental data. Adeq_pre_ ratio for DPPH was > 4, signifying an adequate signal, model efficacy and can be used to navigate the design space. Table 7 shows regression coefficients for DPPH. The positive and negative coefficient values (11.93, −3.39) for DPPH indicates that when MP and MT increased, DPPH values would increase for MP and decrease for MT. The quadratic regression equation describing the effect of the process variables on the DPPH is reported in Table 8. Lastly, optimal parameters (269.7 W, 20 min, 1.01 mm) achieved the maximum DPPH value of 75.61% according to desirability illustrated in Table 9, respectively. Figure 1m presents the standardized Pareto chart, illustrating the linear, quadratic, and interaction effects of each factor studied on the DPPH of persimmon leather. According to this figure, MP is the most significant factor, followed by the MT and LT. Figure 6 illustrates the 3D response surface of moisture content versus MP, MT and LT. According to Figure 6, MP had a greater effect on DPPH than LT and MT. Furthermore, DPPH showed an indirect relation with MT and LT and a direct relation with MP.

## 4 Discussion

### 4.1 Moisture Content

Moisture content decreased with an increase in MP and MT. In contrast, an increase in LT led to higher moisture content. In runs 13 and 10 (Table 4), moisture content dropped significantly. This decrease occurred when MP increased from 90 to 270 W. Extending the drying time from 20 to 60 min led to a significant reduction in moisture content. This was observed in runs 11 and 13. Increasing the LT from 1 to 5 mm caused the moisture content to rise. This was noted in runs 14 and 15. A similar pattern was observed in mango and medlar fruit leather, where MP played a vital role (Pushpa et al. 2006; Suna 2019). The desired moisture content range for the persimmon leather containing *S*_T_ is 15%–18%. This range is consistent with moisture content found in other fruit leathers such as peach, papaya, mango, jamun, apple, and guava. These fruit leathers also typically fall within this moisture range (Torres et al. 2015; Nayaka et al. 2022; Suradkar et al. 2021; Azeredo et al. 2006; Addai et al. 2016; Kumar Jha 2012; Roknul Azam et al. 2019). Lastly, the optimal conditions for achieving a 15% moisture content in persimmon leather, as shown in Table 9, closely align with the experimental values from Table 4. This indicates that high MP and shorter MT effectively meet the desired moisture level.

Factors like *S*_T_, maltodextrin, and guar gum also played a secondary role in reducing moisture content, even though they were added in small amounts. Savita et al. (2004) highlighted that proteins have the capability to enhance the water‐holding capacity of *S*_T_ by improving its swelling ability. The study observed an increase in *S*_T_ water absorption capacity. This improvement may be due to its higher protein content. The complexes of rebaudioside‐A and stevioside with the separating agent demonstrated that both ligands penetrated deeply into the hydrophobic cavity. The presence of additional hydrogen bonding in the case of stevioside is probably responsible for its stronger binding affinity than that of rebaudioside‐A (Ayyappa et al. 2015). *S*_T_ was proposed to have hygroscopic properties due to its stevioside content. Stevioside is a diterpene glycoside consisting of one aglycone molecule (steviol) and three glucose molecules. Glucose molecules have hydroxyl groups (−OH) that could bind water easily and form hydrogen bonds (Belgis et al. 2023). Moreover, maltodextrin and guar gum also played in rapid moisture content loss. Long‐chain molecular structure of guar gum and the abundance of hydroxyl groups in the galactomannan molecule make it fit for a variety of chemical reactions (Thombare et al. 2016). Guar gum is known to form a gel network. This network increases the water‐holding ability (Oliveira et al. 2011). Furthermore, maltodextrin is a humectant due to the presence of hydrophilic group such as hydroxyl groups (Badola et al. 2017).

### 4.2 Color

**FIGURE 7:**
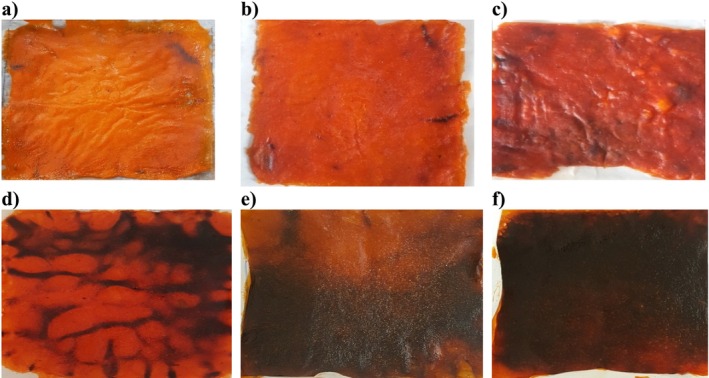
NEB, Maillard reaction, pigment decomposition and caramelization in persimmon leather products containing *S*_T_ (a) 90 W, 20 min, 3 mm; (b) 90 W, 60 min, 3 mm; (c) 180 W, 40 min, 3 mm; (d) 270 W, 20 min, 3 mm; (e) 270 W, 40 min, 5 mm; and (f) 270 W, 60 min, 3 mm.

In color, two distinct patterns were observed. The first pattern demonstrated that increasing MP while maintaining a shorter time‐period resulted in a decrease in L* value and hue angle. This reduction was accompanied by an increase in a*, b*, and chroma. This trend indicates non‐enzymatic browning (NEB) and Maillard reactions. This was observed at 90 W for 20 min and 180 W for 20 min. Increase in a* and decrease in hue angle are prominent indicators of browning. The second pattern pointed toward an extensive breakdown or degradation of pigments. The extensive breakdown involved caramelization and NEB. This phenomenon was notable with prolonged MT at higher MP. For example, at 270 W for 60 min, there was a significant reduction in L*, a*, b*, hue angle, and chroma. A similar reduction was observed at 180 W for 60 min (see Table 4). Similar pigment breakdown and color parameter changes, indicative of non‐enzymatic processes, were observed in hawthorn, peach, pear, pine bark, and cashew apple juice models (Huang and Hsieh 2005; Pu and Sun 2017; Zepka et al. 2009). In the transition from low to high MP, L* values were anticipated to decrease. However, at 270 W for 20 min (Table 4), this expected decrease did not occur. This may be due to the loss of anthocyanins. The increase in L* value indicated a higher depletion of anthocyanins. Persimmons are known for their rich bioactive components like carotenoids, tannins, flavonoids, and anthocyanins. Anthocyanins are highly unstable and easily degraded. In the absence of oxidase enzymes, they degrade and undergo condensation events. This can result in the formation of brown pigment. This pattern was observed in papaya leather, mango, medlar, and mulberry leather. The increase in MP led to a loss of anthocyanins. As a result, the L* value increased (Addai et al. 2016; Pushpa et al. 2006; Suna 2019; Suna and Özkan‐Karabacak 2019). When considering LT, notable reductions in L*, a*, b*, chroma, and hue were observed. For example, in the parameters of 90 W for 20 min at 3 mm thickness and 180 W for 20 min at 1 mm thickness. These observations strongly suggest substantial pigment decomposition or NEB. For the ideal color parameters, except L*, lower MP levels were optimal. Figure 7 presents the degree of browning observed in various parameters. Figure 7a exhibited the least browning. In contrast, Figure 7f displayed the highest degree of browning. This indicates peak pigment degradation, NEB, and caramelization. The degree of browning follows the order of descending a < b < d < c < e < f. To achieve the desired color for *S*_T_ persimmon leather, a high MP level of 270 W for 20 min was necessary for L*. Lower MP levels reduce NEB. This helps in attaining optimal values for a*, b*, chroma, and hue angle. This lower MP also prevents pigment degradation. The optimal values were quite close to experimental values. Both *S*_T_ and maltodextrin contributed in NEB such as Maillard reactions and caramelization in this study. According to Grembecka (Goksel et al. 2013) SGs are stable in high temperature of up to 200°C. *S*_T_ sweetener can be stored for long periods. It is not fermentable and does not undergo a browning reaction. However, microwave irradiation generates intermediate reducing sugars from *S*_T_. The presence of reducing sugars, such as fructose and glucose, is important for developing color through the Maillard reaction. Two main forms of NEB are caramelization and the Maillard reaction (Bharate and Bharate 2014). NEB is produced by caramelization or the Maillard reaction. Caramelization occurs when sucrose is heated above its melting point. In the Maillard reaction, browning is produced by sucrose reacting with amino acid during heating (Tou et al. 2011). Likewise, in the study conducted by De et al. (2013), the content of reducing and non‐reducing sugar in *S*_T_ was determined. The range of reducing sugar was 5.6%–6.1%. Non‐reducing sugar was reported to be in the range of 9.6%–9.9%. Moreover, maltodextrin contains D‐glucose units with α (1 → 4) glycoside bonds. The neutrality and reducing power of maltodextrin chains can facilitate reactions. These reactions occur between maltodextrin and proteins during the Maillard reaction (Nasrollahzadeh et al. 2017). Darkening phenomenon is another cause of the browning during drying (Duan et al. 2016). At low MP and short MT, rapid moisture loss was not possible. As a result, browning did not occur. At higher MP and longer MT, the degree of browning increased. Maillard reactions also became more prominent. This led to greater moisture loss. *S*_T_ also produced intermediate reducing sugars, which contributed to this effect.

### 4.3 Texture

Upon analyzing the results, it became clear that MP was the primary independent variable. It significantly influenced the texture attributes of persimmon leather with *S*_T_. As MP increased from low to high levels, HA, CH, CN, and GU all showed a notable rise. However, SG decreased. This pattern was observed in runs 11 and 9 (Table 4), where MP was raised from 90 W to 270 W. The second variable that had a significant impact on most texture parameters was MT. The observed trend indicated that as the duration of time increased at each power level, HA, CH, GU, and CN increased. Conversely, SG decreased. This pattern was evident in runs 9 and 10 (Table 4), where MT was increased from 20 to 60 min at the same MP. Here, a low value of SG means leather does not come back to its original form once force applied. In the context of papaya leather, prolonged drying periods result in reduced moisture content and a firmer texture (Addai et al. 2016). Similarly, in plum fruit leather, strong positive correlations were observed between HA, CH, and GU (Momchilova et al. 2016). The same trend observed persimmon fruit leather containing *S*_T_ was analogous. As HA increased in persimmon fruit leather, CH, GU, and CN also showed an increase. This pattern was also observed in mulberry leather. An increase in MP resulted in higher values of HA, GM, and CN (Suna and Özkan‐Karabacak 2019). In another study, it was found that higher drying temperatures and increased MP led to reduced moisture content. This reduction in moisture content, in turn, resulted in an increase in CN (Grembecka 2015). In Annona‐based fruit leathers, prolonged drying or higher temperature resulted in a harder texture (Ayalew and Emire 2020). Moisture content significantly influenced the texture attributes. Decreased moisture content is associated with an increase in HA, CH, GU, and CN. However, this decrease in moisture content also leads to a reduction in SG. Lastly, optimal values for HA, CH, CN, GU, and SG were maintained at a moderate level rather than at extremes. These values closely resembled the experimental ones that yielded the most preferred persimmon leather. Under optimal conditions, persimmon leather with *S*_T_ achieved these desired texture values using less MP. Some texture parameters achieved ideal values with higher MP but shorter MT. To achieve the desired texture parameters, either a higher MP with shorter MT or moderate MP levels were necessary. Both approaches maintained the quality of the persimmon leather containing *S*_T_.

### 4.4 Total Phenolic Content

TPC exhibited two discernible patterns. Firstly, TPC demonstrated an increase from low MP to high MP. Secondly, TPC significantly decreased with longer durations at each MP. An analysis of the final TPC values shows an increase in TPC with MP only with a shorter time duration. This is demonstrated in Table 4. This is evident in experimental runs 11 (90 W–20 min) and 9 (270 W–20 min) of persimmon leather with *S*_T_. The rise in TPC was likely caused by the rupture of cellular elements in the persimmon leather. This rupture resulted in a greater release of polyphenolics from the leather texture. Microwave treatment may cleave esterified and glycoside‐bound phenolic acids (Hayat et al. 2010). This could also be linked to the occurrence of NEB or Maillard reactions. Conversely, extending the microwave treatment time caused more extensive structural damage. This affected the cellular components of the persimmon leather. The intense heat facilitated NEB and phenol oxidation processes. Additionally, the decomposition of major persimmon pigments occurred. Consequently, there was a notable reduction in TPC observed in test runs 10 (270 W–60 min) and 9 (270 W–20 min) as detailed in Table 4 for persimmon leather with *S*_T_. Similar pattern was observed in apricot, pomegranate, mulberry leather, citrus mandarin peels, and orange peels (Ghanem et al. 2012; Suna and Özkan‐Karabacak 2019; Suna et al. 2014; Tontul and Topuz 2017). Finally, the objective was to maximize the TPC to optimize the response. The optimized TPC value was achieved under optimal conditions with high MP but a shorter MT in persimmon leather with *S*_T_. The optimized value and conditions were quite similar to the experimental value apart from LT.

Lastly, the minor ingredient *S*_T_ also had an impact on the persimmon leather. *S*_T_ leaf extract contains high levels of total phenolic compounds. It can inhibit and quench free radicals to terminate the radical chain reaction. Additionally, it acts as a reducing agent (De et al. 2013). In the study conducted by Ahmad et al. (2019), they found that stevia extract contains approximately 2.09% ± 0.22% of reducing sugars and about 3.13% ± 0.43% of non‐reducing sugars. This indicates the presence of both reducing and non‐reducing sugars in the stevia aqueous extract. Moreover, SGs have been shown to exert significant stabilities to thermal effects and pH changes (Yılmaz et al. 2021). Similar patterns were observed in this study as well. When MP increased and MT was extended, *S*_T_ produced reducing sugar intermediates. These intermediates contributed to NEB and the Maillard reaction.

### 4.5 2,2‐Diphenyl‐1‐Picrylhydrazyl (DPPH)

Microwave energy can cause structural damage to cells. This damage leads to the production of substances that donate hydrogen atoms. These substances may inhibit free radicals (Tomsone et al. 2020). NEB and pigment degradation were crucial in DPPH, much like in TPC. Both MT and MP played significant roles in persimmon leather with *S*_T_. The trend observed in these leathers mirrored that of TPC. DPPH increased as MP increased and decreased as MP decreased. Likewise, an increase in MT led to a decrease in DPPH. Based on the RSM Box–Behnken design, it was observed that AC increased with higher MP when the duration was kept short. This trend is evident in experimental runs 11 (90 W–20 min) and 9 (270 W–20 min) as shown in Table 4. The increase in AC was attributed to the decomposition or rupture of cellular elements in the persimmon leather. This caused a higher release of polyphenolics from the texture. It was likely due to the Maillard effect. Prolonged MT exposure caused rapid and intense heat. This resulted in more extensive structural damage to the cells. This led to increased NEB and phenol oxidation. Consequently, there was a decrease in AC as well. It was observed in experimental runs 10 (270 W–60 min) and 9 (270 W–20 min) as shown in Table 4 of persimmon leather with *S*_T_. A similar pattern was observed in hawthorn leather, pomegranate leather, mulberry leather, and citrus pomace (Abbaspour‐Gilandeh et al. 2021; Hayat et al. 2010; Suna and Özkan‐Karabacak 2019; Tontul and Topuz 2017). Ultimately, the aim was to maximize the DPPH value to optimize the response. This value was achieved under ideal conditions of high MP and low MT. The optimized value of antioxidant capacity and conditions were quite similar to the experimental value apart from LT.

### 4.6 Comparison and Optimization of Persimmon Leather Produced by Conventional and Microwave Drying

Maintaining a moisture content of around 15% is crucial in fruit leather production. HA, CH, CN, and GU must also be within acceptable levels. Keeping this objective in mind, optimized conditions were analyzed. MP of 210 W, LT of 3.5 mm, and MT of 30 min was selected. Table 10 presents a comparative analysis of drying techniques. It focuses on temperature, duration, thickness, and power for both microwave and hot air methods. The key difference is in the time required. The hot air oven method took 5 h and 30 min. In contrast, the optimized microwave approach achieved results in just 30 min at 210 W MP with an LT of 3.5 mm. Table 11 provides a comprehensive statistical overview of the physicochemical attributes, including their mean values and corresponding standard deviations (SD). It compares microwave and hot air drying. *S*_T_ was used as the sweetener in both methods. The results presented in Table 11 indicate that moisture content and TPC showed statistically significant differences (*p* ≤ 0.05) between the drying methods. In contrast, color, texture parameters, and AC demonstrated highly significant differences (*p* ≤ 0.001). These findings suggest that the drying techniques influenced both moisture content and TPC. The influence on color, texture, and AC was even more substantial. This indicates a prominent variation between the drying methods in terms of their effect on these specific attributes. Consequently, the drying method played a crucial role in determining product quality. The moisture content in the microwave‐dried product was maintained at approximately 15%. In contrast, the hot air oven‐dried product showed a reduction of about 1%. Consequently, this variance influenced essential texture parameters. The hot air oven process showcased elevated texture attributes attributed to its lower moisture content when compared to microwave drying. Notably, hot air drying in mulberry leather at 60°C resulted in notably higher values for hardness, chewiness, and gumminess compared to microwave drying (Suna and Özkan‐Karabacak 2019). Similarly, at the same temperature of 60°C, pomegranate leather prepared through hot air drying showed higher hardness, chewiness, and springiness compared to the product from microwave drying (Tontul and Topuz 2017). On the contrary, prolonged exposure to heat during hot air oven drying led to reduced values in L*, a*, b*, chroma, and hue angle. This significantly altered the color. This color transformation can be attributed to heightened degradation of pigments and NEB reactions. Extended heat exposure adversely affected the physical quality of the hot air‐dried product. It also intensified color changes compared to the microwave‐dried product. Microwave‐dried samples displayed a more yellow and lighter color due to the shorter drying duration. Additionally, microwave drying resulted in higher lightness and yellowness values. This occurred with increased power, as it reduced exposure to Maillard and NEB reactions during drying. The red and yellow color in the product is ascribed to the carotenes presence. Moreover, the increase in a* value may be attributed to pigment decomposition and NEB. Additionally, the lower presence of reducing sugars in stevia and the reducing power of maltodextrin may contribute to reduced NEB reactions. These observed color variations between hot air and microwave drying align with similar findings in hawthorn leather, mulberry leather, medlar fruit leather, and peach leather (Abbaspour‐Gilandeh et al. 2021; Roknul Azam et al. 2019; Suna 2019; Suna and Özkan‐Karabacak 2019). In terms of TPC and AC, the hot air oven‐dried product displayed lower values compared to the microwave‐dried product. The prolonged duration of hot air drying caused extensive cell damage. This led to a significant loss of bioactive compounds and increased oxidation. In contrast, the microwave‐dried product showed higher values for both TPC and DPPH. This increase is attributed to Maillard reactions and the rupture of cellular elements. Phenols are typical substrates for the Maillard reaction. They enhanced TPC and antioxidant activity in the microwave‐dried product (Amaya‐Farfan and Rodriguez‐Amaya 2021). Antioxidant activity results are in line with hawthorn leather, mulberry leather, and pomegranate leather (Abbaspour‐Gilandeh et al. 2021; Suna and Özkan‐Karabacak 2019; Tontul and Topuz 2017).

**TABLE 10:** Optimized conditions for conventional and microwave drying of persimmon leather containing *S*_T_.

Parameters	Hot air oven method	Microwave oven method
Temperature (°C)	60	—
RH (relative humidity) (%)	10	—
Time (min)	330	30
LT (mm)	3.5	3.5
MP (W)	—	210

Abbreviations: LT, leather thickness; MP, microwave power.

**TABLE 11:** Physicochemical attributes of drying techniques on moisture, color, texture, TPC and DPPH of the persimmon leather containing *S*_T_.

Physicochemical attributes	Hot air oven product	Microwave oven optimized product	*p*
	Mean ± SD	Mean ± SD	
Moisture content (%)	14.11 ± 0.19	15.22 ± 0.16	0.002*
L*	30.43 ± 0.51	37.63 ± 0.43	0.000**
a*	22.81 ± 0.32	25.86 ± 0.39	0.000**
b*	20.01 ± 0.11	23.91 ± 0.24	0.000**
Chroma	30.34 ± 0.21	35.22 ± 0.16	0.000**
Hue angle	41.26 ± 0.27	42.76 ± 0.22	0.002*
HA (g)	81.89 ± 1.02	72.77 ± 1.11	0.000**
SG (%)	339.44 ± 2.84	389.86 ± 2.39	0.000**
CH (%)	42.74 ± 0.63	35.33 ± 0.54	0.000**
GU (g)	34.99 ± 0.20	25.71 ± 0.15	0.000**
CN (g)	118.80 ± 1.16	100.23 ± 0.98	0.000**
TPC (mg GAE g^−1^)	0.04 ± 0.01	0.11 ± 0.03	0.025*
DPPH (%)	39.09 ± 0.42	65.43 ± 0.57	0.000**

Abbreviations: a*, redness; b*, yellowness; CH, cohesiveness; CN, chewiness; GU, gumminess; HA, hardness; L*, lightness; NS, non‐significant (> 0.05); SD, standard deviation; SG, springiness; TPC, total phenolic content.

* Significant (≤ 0.05).

** Highly significant (≤ 0.001).

### 4.7 Energy Aspects of Persimmon Leather Containing *S*_T_

Modern drying methods aim to enhance efficiency by minimizing energy consumption and delivering high‐quality results while keeping economic input low (Kassem et al. 2011). The main purpose of energy in the drying process is to remove moisture from food materials. Therefore, energy consumption plays a key role in assessing the overall energy efficiency of the process (Kumar et al. 2022). Hot air convection drying is one of the oldest and most commonly used drying methods. More than 85% of industrial dryers rely on convective hot air drying. However, a major drawback of these dryers is their high energy consumption (Zarein et al. 2015). In contrast, microwave drying offers several benefits, including a faster drying rate, reduced drying time, lower energy consumption, and improved quality of the dried products (Kassem et al. 2011).

The comparison of energy consumption between hot air conventional drying and microwave drying reveals a significant difference in efficiency. The hot air conventional drying method consumes 54.55 kWh of energy, which is substantially higher than the 0.45 kWh consumed by microwave drying as shown in Table 12. Furthermore, the energy efficiency of the drying methods showed notable differences. Hot air drying had an efficiency of 39.71%. In contrast, microwave drying showed a higher efficiency of 55.31%, as demonstrated in Table 12. It used energy more effectively during the drying process. A similar type of pattern energy consumption and energy efficiency between conventional drying had been observed in the drying kinetics and modeling of tomato slices (Guemouni et al. 2022), purple basil leaves (Altay et al. 2019), nettle leaves (Alibas 2007), organic reactions (Moseley and Woodman 2009), hydrogen production from methane dry reforming (Sharifvaghefi et al. 2019), mushroom slices (Motevali, Minaei, Khoshtaghaza, and Amirnejat 2011), and in harm's leaves (Nhu‐Trang et al. 2022).

In hot air drying, heating takes a longer time due to their low thermal conductivity. Microwave drying is more energy‐efficient and consumes less energy than other methods. As MP increases, drying time decreases and the drying rate improves. This happens because bipolar water molecules absorb more energy at higher MP (Motevali, Minaei, Khoshtaghaza, and Amirnejat 2011). This highlights the advantage of microwave drying in terms of energy utilization compared to the conventional hot air method. The drastic difference highlights the energy‐saving potential of microwave drying. It makes it a more efficient option. Consequently, microwave drying reduces both drying time and energy consumption significantly, making it a more sustainable option compared to conventional drying methods. Additionally, it offers a cost‐effective solution due to the lower energy requirements.

### 4.8 Sensory Evaluation

Sensory evaluation holds a pivotal role in the development of novel food items. In the context of this evaluation, the persimmon leather produced under the finalized optimization conditions for microwave drying (210 W, 30 min, 3.5 mm) was compared to hot air oven‐dried leather. Table 13 presents a summary of the statistical analysis of sensory attributes, including sweetness, flavor, stickiness, chewiness, hardness, color, appearance, and overall acceptability. It provides the average mean values along with their corresponding SD. Persimmon leather sweetened with *S*_T_ and dried using the microwave method showed higher average mean scores across all sensory attributes when compared to hot air drying. The statistical analysis revealed that all sensory attributes were highly significant (*p* ≤ 0.001). However, sweetness and stickiness did not exhibit significant differences between the two drying methods. This suggests that the drying method had a stronger impact on other sensory characteristics, but not on sweetness and stickiness. The microwave‐dried leather demonstrated better quality overall. In a similar study on apple and guava leather, microwave drying resulted in superior flavor, color, and taste compared to hot air drying (Bandaru and Bakshi 2021). A radar plot displayed the maximum taste values generated by an electronic tongue for peach leather. The results showed that the microwave‐dried product had a richer taste profile. Additionally, the microwave‐dried leather exhibited more bitterness compared to the hot air oven‐dried product (Roknul Azam et al. 2019). Conversely, in the case of medlar fruit leather, the color, appearance, chewiness, and overall acceptability were found to be better when dried using a hot air oven rather than microwave drying (Suna 2019).

**TABLE 12:** Energy aspects of conventional (hot air oven) and microwave drying methods in persimmon leather products containing *S*_T_.

Drying method	ET (kWh)	*η* (%)
Conventional drying method	54.55	39.71
Microwave drying method	0.45	55.31
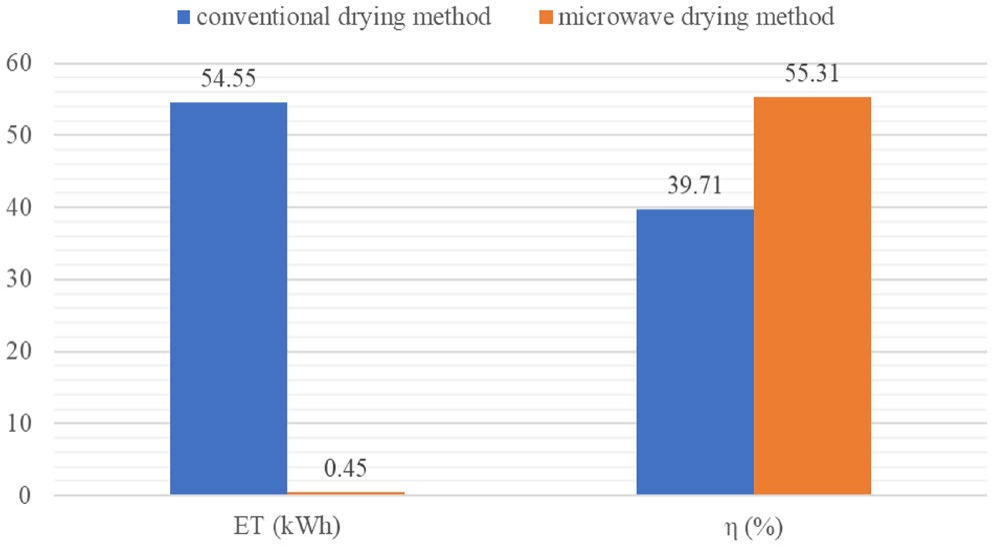		

Abbreviations: (%), energy efficiency; *E*_T_ (kWh), total energy consumption.

**TABLE 13:** Sensory evaluation of the persimmon leather between hot air oven and microwave drying products.

Sensory attributes	Hot air oven product	Microwave oven optimized product	*p*‐value
	Mean ± SD	Mean ± SD	
Sweetness	8.67 ± 0.07	8.73 ± 0.09	0.414
Flavor	7.56 ± 0.07	8.48 ± 0.16	0.001**
Color	7.23 ± 0.13	8.65 ± 0.11	0.000**
Appearance	6.93 ± 0.29	8.52 ± 0.06	0.001**
Hardness	7.04 ± 0.19	8.70 ± 0.11	0.000**
Chewiness	6.86 ± 0.20	8.47 ± 0.12	0.000**
Stickiness	8.36 ± 0.10	8.54 ± 0.09	0.081
Overall acceptability	6.63 ± 0.10	8.69 ± 0.07	0.000**

Abbreviations: NS, non‐significant (> 0.05); SD, standard deviation.

*Note:* *Significant (≤ 0.05); **Highly significant (≤ 0.001); NS = non‐significant (> 0.05) where SD: Standard deviation.

## 5 Conclusions

In this study, persimmon leather production was explored using *S*_T_ as a sweetener and a variety of hydrocolloids. Guar gum exhibited superior textural properties in comparison to corn starch and pectin. *S*_T_ was retained as a constant ingredient. Both *S*_T_ and guar gum were processed further for the final trials. Microwave drying was optimized with the RSM Box–Behnken design. The independent variables were MP, MT, and LT. Reducing sugar intermediates generation, water‐holding properties of *S*_T_ during microwave heating, and reducing power of maltodextrin significantly influenced moisture content, texture, color, TPC, and antioxidant activity (DPPH). Higher MP and MT also promoted Maillard reactions, NEB, pigment decomposition, and moisture reduction. The optimization approach highlighted distinct characteristics of persimmon leather sweetened with *S*_T_. It emphasized the individual traits of each sample. Two strategies were used. One involved low MP with high MT, and the other used high MP with low MT. The microwave drying method produced a better‐quality product and improved physicochemical properties. It also required less time than the traditional hot air oven drying. These findings provide important insights for improving persimmon leather quality through microwave drying. This method focused on enhancing energy efficiency. In contrast, hot air drying showed high energy consumption and low efficiency. Microwave drying, on the other hand, had higher energy efficiency and consumed less energy. Sensory evaluation underscored the superiority of *S*_T_ sweetened optimized product from the microwave. It showcased better overall acceptability, flavor, chewiness, hardness, appearance, and color compared to conventionally dried leather product. Consequently, it is recommended to embrace novel microwave‐based processing technology to uphold the superior quality of persimmon leather products. Future research should look into optimizing microwave drying to further boost energy efficiency and maintain the quality of persimmon leather. Testing different hydrocolloids with *S*_T_ could help enhance texture and taste. It is also important to study how well microwave‐dried leather keeps its quality and nutrients over time. Exploring how this method can be scaled up for large‐scale production and applied to other fruits would be valuable. Additionally, investigating the cost‐effectiveness and potential environmental impacts of the microwave drying process could provide further insights.

## Author Contributions

**Muhammad Hamza Alam:** conceptualization (equal), data curation (equal), formal analysis (equal), supervision (equal), validation (equal), visualization (equal). **Muhammad Haseeb Ahmad:** funding acquisition (equal), investigation (equal), supervision (equal), visualization (equal), writing – original draft (equal). **Muhammad Imran:** funding acquisition (equal), methodology (equal), project administration (equal), resources (equal), software (equal), validation (equal), writing – review and editing (equal). **Misbah Ur Rehman:** conceptualization (equal), data curation (equal), formal analysis (equal), methodology (equal), writing – original draft (equal). **Muhammad Imran Khan:** data curation (equal), formal analysis (equal), funding acquisition (equal), project administration (equal), validation (equal), visualization (equal), writing – original draft (equal). **Muhammad Kamran Khan:** conceptualization (equal), data curation (equal), investigation (equal), supervision (equal), validation (equal), writing – original draft (equal). **Waseem Khalid:** conceptualization (equal), data curation (equal), methodology (equal), software (equal), visualization (equal), writing – review and editing (equal). **Sulaiman Ali Alharbi:** funding acquisition (equal), investigation (equal), methodology (equal), supervision (equal), validation (equal), writing – review and editing (equal). **Hossam M. Aljawdah:** data curation (equal), funding acquisition (equal), methodology (equal), software (equal), validation (equal), writing – original draft (equal). **Felix Kwashie Madilo:** resources (equal), software (equal), supervision (equal), validation (equal), visualization (equal), writing – original draft (equal), writing – review and editing (equal).

## Conflicts of Interest

The authors declare no conflicts of interest.

## Data Availability

All the data that support the findings of this study are present in the article.

## References

[fsn370036-bib-0001] Abbaspour‐Gilandeh, Y. , M. Kaveh , H. Fatemi , and M. Aziz . 2021. “Combined Hot Air, Microwave, and Infrared Drying of Hawthorn Fruit: Effects of Ultrasonic Pretreatment on Drying Time, Energy, Qualitative, and Bioactive Compounds' Properties.” Food 10: 1006. 10.3390/foods10051006 PMC8147953 34064476

[fsn370036-bib-0002] Acar, C. , I. Dincer , and A. Mujumdar . 2022. “A Comprehensive Review of Recent Advances in Renewable‐Based Drying Technologies for a Sustainable Future.” Drying Technology 40: 1029–1050.

[fsn370036-bib-0003] Addai, Z. R. , A. Abdullah , S. A. Mutalib , and K. H. Musa . 2016. “Evaluation of Fruit Leather Made From Two Cultivars of Papaya.” Italian Journal of Food Science 28: 73–82.

[fsn370036-bib-0004] Ahmad, U. , R. S. Ahmad , A. Imran , Z. Mushtaqq , and S. M. Hussian . 2019. “Characterization of Low Calorie Ready‐To‐Serve Peach Beverage Using Natural Sweetener, Stevia (*Stevia rebaudiana* bertoni).” Journal of Nutrition and Internal Medicine 21: 435–444.

[fsn370036-bib-0005] Alibas, I. 2007. “Energy Consumption and Color Characteristics of Nettle Leaves During Microwave, Vacuum and Convective Drying.” Biosystems Engineering 96, no. 4: 495–502.

[fsn370036-bib-0006] Altay, K. , A. A. Hayaloglu , and S. N. Dirim . 2019. “Determination of the Drying Kinetics and Energy Efficiency of Purple Basil (*Ocimum basilicum* L.) Leaves Using Different Drying Methods.” Heat and Mass Transfer 55: 2173–2184.

[fsn370036-bib-0007] Amaya‐Farfan, J. , and D. B. Rodriguez‐Amaya . 2021. “The Maillard Reactions.” In Chemical Changes During Processing and Storage of Foods, 215–263. Elsevier.

[fsn370036-bib-0008] Ayalew, G. M. , and S. A. Emire . 2020. “Formulation and Characterization of Fruit Leather Based on *Annona muricata* l. Fruit and *Avena sativa* Flour.” Journal of Food Processing and Preservation 44: e14284.

[fsn370036-bib-0009] Ayyappa, B. , S. Kanchi , P. Singh , M. I. Sabela , M. Dovey , and K. Bisetty . 2015. “Analytical Evaluation of Steviol Glycosides by Capillary Electrophoresis Supported With Molecular Docking Studies.” Journal of the Iranian Chemical Society 12: 127–136.

[fsn370036-bib-0010] Azeredo, H. M. , E. S. Brito , G. E. Moreira , V. L. Farias , and L. M. Bruno . 2006. “Effect of Drying and Storage Time on the Physico‐Chemical Properties of Mango Leathers.” International Journal of Food Science and Technology 41: 635–638.

[fsn370036-bib-0011] Badola, R. , R. R. B. Singh , N. R. Panjagari , A. K. Singh , and S. A. Hussain . 2017. “Effect of Selected Humectants as Water Activity Modifiers on the Quality of Model Khoa System.” Indian Journal of Dairy Science 70: 145–154.

[fsn370036-bib-0012] Bandaru, H. , and M. Bakshi . 2021. “Effect of Different Drying Conditions on the Quality of Apple and Guava Fruit Leather.” Pharma Innovation Journal 10, no. 8: 233–237.

[fsn370036-bib-0013] Beigi, M. 2016. “Energy Efficiency and Moisture Diffusivity of Apple Slices During Convective Drying.” Food Science and Technology (Campinas) 36, no. 1: 145–150.

[fsn370036-bib-0014] Belgis, M. , A. D. Masahid , F. A. Rahmawati , and N. F. Sadek . 2023. “Antioxidant, Anti‐Microbial, and Physical Properties Improvement of Turmeric (*Curcuma domestica* val.) Effervescent Tablets With Stevia (Stevia Rebaudiana) Leaf Powder, AIP Conference Proceedings.” AIP Publishing.

[fsn370036-bib-0015] Bharate, S. S. , and S. B. Bharate . 2014. “Non‐Enzymatic Browning in Citrus Juice: Chemical Markers, Their Detection and Ways to Improve Product Quality.” Journal of Food Science and Technology 51: 2271–2288. 25328169 10.1007/s13197-012-0718-8 PMC4190239

[fsn370036-bib-0016] Chibuike, O. , D. O. Chukwudozie , D. N. Reginald , D. C. Anthony , D. O. Johnson , and P. E. Anyanwu . 2021. “Energy Consumption of Yam Slice Drying in an Exhaust Gas Waste Heat Recovery Hot Air Tray Dryer.” Scientific Research Journal 9, no. 8: 1–7.

[fsn370036-bib-0017] Concha‐Meyer, A. A. , V. D'Ignoti , B. Saez , R. I. Diaz , and C. A. Torres . 2016. “Effect of Storage on the Physico‐Chemical and Antioxidant Properties of Strawberry and Kiwi Leathers.” Journal of Food Science 81: C569–C577. 26799705 10.1111/1750-3841.13214

[fsn370036-bib-0018] Das, K. , M. Kumar , and A. Das . 2019. “Standardization of Packaging Material and Storage Condition for Pomegranate Leather.” International Journal of Current Microbiology and Applied Sciences 8: 2748–2760.

[fsn370036-bib-0019] De, S. , S. Mondal , and S. Banerjee . 2013. Stevioside: Technology, Applications and Health. John Wiley & Sons.

[fsn370036-bib-0020] Duan, X. , W. C. Liu , G. Y. Ren , L. L. Liu , and Y. H. Liu . 2016. “Browning Behavior of Button Mushrooms During Microwave Freeze‐Drying.” Drying Technology 34: 1373–1379.

[fsn370036-bib-0021] Dursun, D. , and A. C. Dalgıç . 2018. “Production and Preference Mapping of Persimmon Fruit Leather: An Optimization Study by Box–Behnken.” Journal of Food Process Engineering 41: e12899.

[fsn370036-bib-0022] Flieger, J. , and M. Flieger . 2020. “The [Dpph●/Dpph‐h]‐Hplc‐Dad Method on Tracking the Antioxidant Activity of Pure Antioxidants and Goutweed ( *Aegopodium podagraria* l.) Hydroalcoholic Extracts.” Molecules 25: 6005. 33353137 10.3390/molecules25246005 PMC7766071

[fsn370036-bib-0023] Gámbaro, A. , and M. B. McSweeney . 2020. “Chapter Eight ‐ Sensory Methods Applied to the Development of Probiotic and Prebiotic Foods.” In Advances in Food and Nutrition Research, edited by A. G. da Cruz , E. S. Prudencio , E. A. Esmerino , and M. C. da Silva , vol. 94, 295–337. Academic Press. 10.1016/bs.afnr.2020.06.006 32892836

[fsn370036-bib-0024] Ghanem, N. , D. Mihoubi , N. Kechaou , and N. B. Mihoubi . 2012. “Microwave Dehydration of Three Citrus Peel Cultivars: Effect on Water and Oil Retention Capacities, Color, Shrinkage and Total Phenols Content.” Industrial Crops and Products 40: 167–177.

[fsn370036-bib-0025] Goksel, M. , M. Dogan , O. S. Toker , S. Ozgen , K. Sarioglu , and R. A. Oral . 2013. “The Effect of Starch Concentration and Temperature on Grape Molasses: Rheological and Textural Properties.” Food and Bioprocess Technology 6: 259–271.

[fsn370036-bib-0026] Grembecka, M. 2015. “Natural Sweeteners in a Human Diet.” Roczniki Państwowego Zakładu Higieny 66: 195–202. 26400114

[fsn370036-bib-0027] Guemouni, S. , K. Mouhoubi , F. Brahmi , F. Dahmoune , A. Belbahi , and C. Benyoub . 2022. “Convective and Microwave Drying Kinetics and Modeling of Tomato Slices, Energy Consumption, and Efficiency.” Journal of Food Process Engineering 45, no. 9: e14113.

[fsn370036-bib-0028] Hayat, K. , X. Zhang , U. Farooq , et al. 2010. “Effect of Microwave Treatment on Phenolic Content and Antioxidant Activity of Citrus Mandarin Pomace.” Food Chemistry 123: 423–429.

[fsn370036-bib-0029] Huang, X. , and F. H. Hsieh . 2005. “Physical Properties, Sensory Attributes, and Consumer Preference of Pear Fruit Leather.” Journal of Food Science 70: E177–E186.

[fsn370036-bib-0030] Karp, S. , J. Wyrwisz , M. Kurek , and A. Wierzbicka . 2016. “Physical Properties of Muffins Sweetened With Steviol Glycosides as the Sucrose Replacement.” Food Science and Biotechnology 25: 1591–1596. 30263449 10.1007/s10068-016-0245-x PMC6049254

[fsn370036-bib-0031] Kassem, A. , A. Shokr , A. El‐Mahdy , A. Aboukarima , and E. Hamed . 2011. “Comparison of Drying Characteristics of Thompson Seedless Grapes Using Combined Microwave Oven and Hot Air Drying.” Journal of the Saudi Society of Agricultural Sciences 10, no. 1: 33–40.

[fsn370036-bib-0032] Khan, M. A. , M. Azam , S. Ahmad , and M. Atiq . 2023. “Improvement of Physicochemicals, Antioxidant System and Softening Enzymes by Postharvest l‐Arginine Application Leads to Maintain Persimmon Fruit Quality Under Low Temperature Storage.” Journal of Food Measurement and Characterization 17: 2964–2977.

[fsn370036-bib-0033] Ko, J.‐A. , Y.‐B. Ryu , W.‐S. Lee , K. Ameer , and Y.‐M. Kim . 2021. “Optimization of Microwave‐Assisted Green Method for Enhanced Solubilization of Water‐Soluble Curcuminoids Prepared Using Steviol Glycosides.” Food 10: 2803. 10.3390/foods10112803 PMC8619202 34829084

[fsn370036-bib-0034] Kumar, A. , P. Kandasamy , I. Chakraborty , and L. Hangshing . 2022. “Analysis of Energy Consumption, Heat and Mass Transfer, Drying Kinetics and Effective Moisture Diffusivity During Foam‐Mat Drying of Mango in a Convective Hot‐Air Dryer.” Biosystems Engineering 219: 85–102.

[fsn370036-bib-0035] Kumar Jha, Y. 2012. “Optimization of Ingredients Level in Low Calorie‐High Protein Papaya Fruit Bar Using Response Surface Methodology.” Journal of Food Processing and Technology 3: 1–5.

[fsn370036-bib-0036] Menon, A. , V. Stojceska , and S. A. Tassou . 2020. “A Systematic Review on the Recent Advances of the Energy Efficiency Improvements in Non‐Conventional Food Drying Technologies.” Trends in Food Science and Technology 100: 67–76.

[fsn370036-bib-0037] Mimica‐Dukic, N. , B. Bozin , M. Sokovic , and N. Simin . 2004. “Antimicrobial and Antioxidant Activities of *Melissa officinalis* l. (Lamiaceae) Essential Oil.” Journal of Agricultural and Food Chemistry 52: 2485–2489. 15113145 10.1021/jf030698a

[fsn370036-bib-0038] Mohamed, A. H. A. , M. Ragab , H. A. I. Siliha , and L. A. M. M. Haridy . 2018. “Physicochemical, Microbiological and Sensory Characteristics of Persimmon Fruit Leather.” Zagazig Journal of Agricultural Research 45: 2071–2085.

[fsn370036-bib-0039] Momchilova, M. , G. Zsivanovits , I. Milkova‐Tomova , D. Buhalova , and P. Dojkova . 2016. “Sensory and Texture Characterisation of Plum (*Prunus domestica*) Fruit Leather.” Bulgarian Chemical Communications 48: 428–434.

[fsn370036-bib-0040] Moseley, J. D. , and E. K. Woodman . 2009. “Energy Efficiency of Microwave‐and Conventionally Heated Reactors Compared at Meso Scale for Organic Reactions.” Energy and Fuels 23, no. 11: 5438–5447.

[fsn370036-bib-0041] Motevali, A. , S. Minaei , and M. H. Khoshtagaza . 2011. “Evaluation of Energy Consumption in Different Drying Methods.” Energy Conversion and Management 52, no. 2: 1192–1199.

[fsn370036-bib-0042] Motevali, A. , S. Minaei , M. H. Khoshtaghaza , and H. Amirnejat . 2011. “Comparison of Energy Consumption and Specific Energy Requirements of Different Methods for Drying Mushroom Slices.” Energy 36, no. 11: 6433–6441.

[fsn370036-bib-0043] Nasr, F. , F. Razavi , V. Rabiei , G. Gohari , S. Ali , and C. Hano . 2022. “Attenuation of Chilling Injury and Improving Antioxidant Capacity of Persimmon Fruit by Arginine Application.” Food 11: 2419. 10.3390/foods11162419 PMC9407207 36010419

[fsn370036-bib-0044] Nasrollahzadeh, F. , M. Varidi , A. Koocheki , and F. Hadizadeh . 2017. “Effect of Microwave and Conventional Heating on Structural, Functional and Antioxidant Properties of Bovine Serum Albumin‐Maltodextrin Conjugates Through Maillard Reaction.” Food Research International 100: 289–297. 28888453 10.1016/j.foodres.2017.08.030

[fsn370036-bib-0045] Nayaka, V. K. , R. Tiwari , C. Narayana , et al. 2022. “Comparative Effect of Different Sugars Instigating Non‐Enzymatic Browning and Maillard Reaction Products in Guava Fruit Leather.” Journal of Horticultural Sciences 17: 174–183.

[fsn370036-bib-0046] Nhu‐Trang, T.‐T. , T.‐K.‐T. Phan , T.‐M.‐T. Lam , P.‐B.‐D. Nguyen , and T.‐V.‐L. Nguyen . 2022. “Drying Kinetics and Energy Consumption in Hot‐Air and Microwave Drying of *Polyscias fruticosa* (L.) Harms Leaves.” Paper presented at the AIP Conference Proceedings.

[fsn370036-bib-0047] Oliveira, N. M. , F. Q. Dourado , A. M. Peres , M. V. Silva , J. M. Maia , and J. A. Teixeira . 2011. “Effect of Guar Gum on the Physicochemical, Thermal, Rheological and Textural Properties of Green Edam Cheese.” Food and Bioprocess Technology 4: 1414–1421.

[fsn370036-bib-0048] Pérez‐Burillo, S. , M. J. Oliveras , J. Quesada , J. A. Rufián‐Henares , and S. Pastoriza . 2018. “Relationship Between Composition and Bioactivity of Persimmon and Kiwifruit.” Food Research International 105: 461–472. 29433237 10.1016/j.foodres.2017.11.022

[fsn370036-bib-0049] Pu, Y.‐Y. , and D.‐W. Sun . 2017. “Combined Hot‐Air and Microwave‐Vacuum Drying for Improving Drying Uniformity of Mango Slices Based on Hyperspectral Imaging Visualisation of Moisture Content Distribution.” Biosystems Engineering 156: 108–119.

[fsn370036-bib-0050] Pushpa, G. , P. Rajkumar , Y. Gariepy , and G. Raghavan . 2006. “Microwave Drying of Enriched Mango Fruit Leather.” ASAE Annual Meeting, 2006; American Society of Agricultural and Biological Engineers: p 1.

[fsn370036-bib-0051] Rodriguez Furlán, L. T. , Y. A. Baracco , N. E. Zaritzky , and M. E. Campderrós . 2016. “Development of Free Sugar White Chocolate, Suitable for Diabetics, Using Stevia and Sucralose as Sweeteners: Study of the Thermal Degradation Kinetic.” International Journal of Research in Advent Technology 4, no. 7: 49–57.

[fsn370036-bib-0052] Roknul Azam, S. , M. Zhang , C. L. Law , and A. S. Mujumdar . 2019. “Effects of Drying Methods on Quality Attributes of Peach ( *Prunus persica* ) Leather.” Drying Technology 37: 341–351.

[fsn370036-bib-0053] Savita, S. , K. Sheela , S. Sunanda , A. Shankar , and P. Ramakrishna . 2004. “ *Stevia rebaudiana*–a Functional Component for Food Industry.” Journal of Human Ecology 15: 261–264.

[fsn370036-bib-0054] Sharifvaghefi, S. , B. Shirani , M. Eic , and Y. Zheng . 2019. “Application of Microwave in Hydrogen Production From Methane Dry Reforming: Comparison Between the Conventional and Microwave‐Assisted Catalytic Reforming on Improving the Energy Efficiency.” Catalysts 9, no. 7: 618.

[fsn370036-bib-0055] Singleton, V. L. , and J. A. Rossi . 1965. “Colorimetry of Total Phenolics With Phosphomolybdic‐Phosphotungstic Acid Reagents.” American Journal of Enology and Viticulture 16: 144–158.

[fsn370036-bib-0056] Soysal, Y. , S. Öztekin , and Ö. Eren . 2006. “Microwave Drying of Parsley: Modelling, Kinetics, and Energy Aspects.” Biosystems Engineering 93, no. 4: 403–413.

[fsn370036-bib-0057] Sun, J. , W. Wang , and Q. Yue . 2016. “Review on Microwave‐Matter Interaction Fundamentals and Efficient Microwave‐Associated Heating Strategies.” Materials 9: 231. 28773355 10.3390/ma9040231 PMC5502878

[fsn370036-bib-0058] Suna, S. 2019. “Effects of Hot Air, Microwave and Vacuum Drying on Drying Characteristics and In Vitro Bioaccessibility of Medlar Fruit Leather (Pestil).” Food Science and Biotechnology 28: 1465–1474. 31695945 10.1007/s10068-019-00588-7 PMC6811487

[fsn370036-bib-0059] Suna, S. , and A. Özkan‐Karabacak . 2019. “Investigation of Drying Kinetics and Physicochemical Properties of Mulberry Leather (Pestil) Dried With Different Methods.” Journal of Food Processing and Preservation 43: e14051.

[fsn370036-bib-0060] Suna, S. , C. E. Tamer , B. Inceday , G. Ö. Sinir , and Ö. U. Çopur . 2014. “Impact of Drying Methods on Physicochemical and Sensory Properties of Apricot Pestil.” Indian Journal of Traditional Knowledge 13, no. 1: 47–55.

[fsn370036-bib-0061] Suradkar, N. , V. Pawar , H. Deshpande , V. Mane , J. Ughade , and A. Ghorband . 2021. “Storage Stability of Jamun Fruit Bar With Respect to Different Temperature and Packaging Material.” Pharma Innovation 10: 1172–1176.

[fsn370036-bib-0062] Surendhar, A. , V. Sivasubramanian , D. Vidhyeswari , and B. Deepanraj . 2019. “Energy and Exergy Analysis, Drying Kinetics, Modeling and Quality Parameters of Microwave‐Dried Turmeric Slices.” Journal of Thermal Analysis and Calorimetry 136: 185–197.

[fsn370036-bib-0063] Thombare, N. , U. Jha , S. Mishra , and M. Siddiqui . 2016. “Guar Gum as a Promising Starting Material for Diverse Applications: A Review.” International Journal of Biological Macromolecules 88: 361–372. 27044346 10.1016/j.ijbiomac.2016.04.001

[fsn370036-bib-0064] Tomsone, L. , R. Galoburda , Z. Kruma , and I. Cinkmanis . 2020. “Characterization of Dried Horseradish Leaves Pomace: Phenolic Compounds Profile and Antioxidant Capacity, Content of Organic Acids, Pigments and Volatile Compounds.” European Food Research and Technology 246: 1647–1660.

[fsn370036-bib-0065] Tontul, I. , and A. Topuz . 2017. “Effects of Different Drying Methods on the Physicochemical Properties of Pomegranate Leather (Pestil).” LWT ‐ Food Science and Technology 80: 294–303.

[fsn370036-bib-0066] Torres, C. A. , L. A. Romero , and R. I. Diaz . 2015. “Quality and Sensory Attributes of Apple and Quince Leathers Made Without Preservatives and With Enhanced Antioxidant Activity.” LWT ‐ Food Science and Technology 62: 996–1003.

[fsn370036-bib-0067] Tou, J. , C. Fitch , and K. Bridges . 2011. “Sweeteners: Uses, Dietary Intake and Health Effects.” In Chocolate, Fast Foods and Sweeteners: Consumption and Health, edited by M. R. Bishop , 1–28. Nova Science Publishers, Inc.

[fsn370036-bib-0068] Vieira, M. , L. Estrella , and S. Rocha . 2007. “Energy Efficiency and Drying Kinetics of Recycled Paper Pulp.” Drying Technology 25, no. 10: 1639–1648.

[fsn370036-bib-0069] Wu, G. , C. F. Morris , and K. M. Murphy . 2014. “Evaluation of Texture Differences Among Varieties of Cooked Quinoa.” Journal of Food Science 79: S2337–S2345. 25308337 10.1111/1750-3841.12672

[fsn370036-bib-0070] Yang, Y. , M. Xu , Z. Wan , and X. Yang . 2022. “Novel Functional Properties and Applications of Steviol Glycosides in Foods.” Current Opinion in Food Science 43: 91–98.

[fsn370036-bib-0072] Yılmaz, F. M. , A. Görgüç , Ö. Uygun , and C. Bircan . 2021. “Steviol Glycosides and Polyphenols Extraction From *Stevia Rebaudiana* Bertoni Leaves Using Maceration, Microwave‐, and Ultrasound‐Assisted Techniques.” Separation Science and Technology 56: 936–948.

[fsn370036-bib-0073] Yilmaz, P. , E. Demirhan , and B. Özbek . 2021. “Microwave Drying Effect on Drying Characteristic and Energy Consumption of *Ficus carica* Linn Leaves.” Journal of Food Process Engineering 44, no. 10: e13831.

[fsn370036-bib-0074] Zarein, M. , S. H. Samadi , and B. Ghobadian . 2015. “Investigation of Microwave Dryer Effect on Energy Efficiency During Drying of Apple Slices.” Journal of the Saudi Society of Agricultural Sciences 14, no. 1: 41–47.

[fsn370036-bib-0075] Zepka, L. Q. , C. D. Borsarelli , M. A. A. P. da Silva , and A. Z. Mercadante . 2009. “Thermal Degradation Kinetics of Carotenoids in a Cashew Apple Juice Model and Its Impact on the System Color.” Journal of Agricultural and Food Chemistry 57: 7841–7845. 19663479 10.1021/jf900558a

